# Simple model of complex dynamics of activity patterns in developing networks of neuronal cultures

**DOI:** 10.1371/journal.pone.0218304

**Published:** 2019-06-27

**Authors:** Ivan Y. Tyukin, Dmitriy Iudin, Feodor Iudin, Tatiana Tyukina, Victor Kazantsev, Irina Mukhina, Alexander N. Gorban

**Affiliations:** 1 Nizhny Novgorod State University, Nizhny Novgorod, Russia; 2 Institute of Applied Physics of RAS, Nizhny Novgorod, Russia; 3 Saint-Petersburg State Electrotechnical University (LETI), Saint-Petersburg, Russia; 4 University of Leicester, Leicester, United Kingdom; Georgia State University, UNITED STATES

## Abstract

Living neuronal networks in dissociated neuronal cultures are widely known for their ability to generate highly robust spatiotemporal activity patterns in various experimental conditions. Such patterns are often treated as neuronal avalanches that satisfy the power scaling law and thereby exemplify self-organized criticality in living systems. A crucial question is how these patterns can be explained and modeled in a way that is biologically meaningful, mathematically tractable and yet broad enough to account for neuronal heterogeneity and complexity. Here we derive and analyse a simple network model that may constitute a response to this question. Our derivations are based on few basic phenomenological observations concerning the input-output behavior of an isolated neuron. A distinctive feature of the model is that at the simplest level of description it comprises of only two variables, the network activity variable and an exogenous variable corresponding to energy needed to sustain the activity, and few parameters such as network connectivity and efficacy of signal transmission. The efficacy of signal transmission is modulated by the phenomenological energy variable. Strikingly, this simple model is already capable of explaining emergence of network spikes and bursts in developing neuronal cultures. The model behavior and predictions are consistent with published experimental evidence on cultured neurons. At the larger, cellular automata scale, introduction of the energy-dependent regulatory mechanism results in the overall model behavior that can be characterized as balancing on the edge of the network percolation transition. Network activity in this state shows population bursts satisfying the scaling avalanche conditions. This network state is self-sustainable and represents energetic balance between global network-wide processes and spontaneous activity of individual elements.

## Introduction

Exploiting physics’ concepts for dealing with problems in life sciences is a widely recognized and successful strategy for developing systematic and lawful understanding of complex phenomena observed in empirical data. One of the most striking and fashionable illustrations facilitating potential and power of this approach is the well-known example of using the concept of self-organized criticality (SOC)—the ability of systems to selftune to the critical state—for explaining a number of puzzling effects in biological systems. Initially proposed as a model for explaining how an abstract system can remain at a critical state in presence of perturbations [1, 2], the concept is now broadly used for describing biological neural networks (see e.g. [3, 4]). It was shown that adaptively evolving networks, i.e., networks combining structural evolution of the network topology with dynamics in the network nodes [5], can exhibit highly robust global SOC-like behavior maintained by simple local network adjustment rules.

Striking examples of SOC-like behavior have been found in experimental studies of neuronal cultures [6–8], with potential benefits of criticality in neural systems discussed in [9], [10]. The cultures grow autonomously and form synaptically coupled networks of living cells. After a period of initial growth and development the cultures start to generate spontaneous activity patterns in the form of population bursts. These bursts are shown to satisfy the power scaling law and hence are often referred as neuronal avalanches [6, 7].

Since then a number of mathematical models have been proposed for simulation and analysis of spontaneous burst generation in neuronal networks. The spectrum of network’s features linked to emergence of persistent bursts includes, but is not limited to re-wiring, delays [11, 12], cycles [13], [14], frequency dependent and spike timing dependent synaptic plasticity [15–17]. With regards to the mathematical frameworks describing neuronal avalanches, models of network’s growth [18] and stochastic networks [19] have been put forward. These results advance our understanding of the phenomenon substantially; yet macroscopic physical mechanisms (expressed e.g. in terms of energy balances) that are responsible for steering living neuronal networks to the burst multiscale dynamics are still unclear.

It has been shown recently (see e.g. [20] and references therein) that population spikes and bursts can be attributed to cells’ adaptation and short time plasticity mechanisms. The authors showed that population spikes, that are similar to the ones observed in in-vitro cultures, can occur in networks of excitatory model neurons with leaky integrate and fire dynamics. These neurons where subjected to Gaussian white noise and equipped with adaptation and short term plasticity mechanism. The network connectivity was all-to-all. Effect of growing neuronal connectivity on bursts was studied in [21]. It has been found that network connectivity expressed e.g. as the number of synaptic connections per neuron, may play an important role for spiking and bursting activity in cultures. Additional links between connectivity development, firing activity homeostasis, and criticality are exposed in [22].

In this work we further contribute to the idea that several features of complex and critical behavior (e.g. the neuronal avalanches, super-bursts, periodic and chaotic spiking) observed in live neuronal cultures and networks can be explained by just few variables. These variables can be linked to local connectivity patterns (expressed e.g. by connection densities between cells) and neuronal activation dynamics.

We demonstrate that main critical transitions can be captured by a hierarchy of simple models. Starting from elementary phenomenological description of neural firing we present a simple 2*D* mean-field model that is capable of showing a broad range of behaviors that are observed in cultures. Remarkably, the network connectivity parameter appears to be a natural bifurcation parameter of the model; it regulates emergence of activity spikes from the initial silent mode to bursts to whole-network activation. This is in good agreement with previous works on SOC phenomena in developing neuronal networks [23], [22]. In contrast to [23], [22], however, we consider the problem from a different angle. Instead of focusing on the activity-connectivity interplay we consider and analyze the system dynamics in the activity-energy plane for various values of connectivity. This adds additional modelling capabilities with regards to investigating effects of oxygen and energy deprivation on neuronal networks behavior whilst retaining important links between network activity and connectivity. Moving further to multi-agent model reveals emergence of neuronal avalanches showing scale-free activity.

The manuscript is organized as follows. In Results we present ingredients of the model at three different levels of phenomenological detail. We begin with a simple percolation-based geometric model describing the evolution of cells’ connectivity. The model allows to accommodate biologically relevant features such as axons and dendrites; it also enables to replicate directional connectivity that is inherent to living systems including neuronal cultures. The model analysis reveals that sharp changes in the overall clustering and connectivity of the evolving network, in both directed and undirected settings, is determined by a single parameter describing average connection density in the network. The effect is qualitatively consistent with empirical evidence reported e.g. in [24]. The analysis is followed by expressing dynamics of neuronal activity by a mean-field approximation. We show that the corresponding single-dimensional model does not explain network spikes and bursts frequently observed in developing cultures. This limitation, however, can be resolved if neural activation is linked to an additional exogenous regulatory energy variable. Introduction of the latter variable needs an additional comment. It behaves as a sort of “energy” or a resource. Its physical nature, nevertheless, may or may not be associated with a specific type of the physical energy. A prototype of such energy, the notion of *adaptation energy*, was introduced by Selye in his analysis of physiological adaption [25], [26] and was successfully employed in modelling of various complex phenomena [27], [28]. The extended model, which may also be viewed as a resource-limited model [29], [30], is shown to be able to reproduce periodic spiking, irregular dynamics, and population bursts observed in live cultures. What is important is that dynamic regimes exhibited by the model can be regulated by just a single parameter corresponding to network connectivity. Next we provide results of large-scale simulation of evolving network of agents of which the activation probability depends on their current energy level. The network dynamics in this state shows population bursts satisfying the scaling avalanche conditions. This network state is self-sustainable and represents energetic balance between global network-wide processes and spontaneous activity of individual elements. The results are then compared with published empirical data. Details of numerical experiments are provided in Materials and Methods.

## Results

### Model

#### Geometric model

We start with a geometrical arrangement of the network elements. Consider a network of *N* neurons whose spatial coordinates are randomly and uniformly distributed in the unit square. Each individual neuron is described by two basic elements. The first, is the region of reception of inputs represented by a circle of a given radius *R*. The circle models neuron’s ability to sense input signals from other neurons, and is referred to as the dendrite region (in biology, dendrite is an input). The second element is an axon (in biology, output), which in our model is simulated by a straight segment of length *H* (on the mature stage of the network development *H* > *R*) and whose end point is acting as a transmitter of the neuron’s signal. If this point reaches out to the dendrite region of another neuron, a connection is established between these neurons [18]. There are three different ways that yield geometrical coupling or connectivity of the network elements:

*Case 1*: cells without axons, i.e. *H* = 0. In this case *N* circles with radius *R* are randomly and uniformly distributed in the unit square. If a circle **A** overlaps with a circle **B**, and circle **B** is connected with a circle **C**, then **A** is connected with **C**. Thus, a path between two distant cells can be defined as a chain of overlapping circles joining these cells. Emergence of large groups of connected elements in this network can be analyzed within the framework of standard circle percolation problem. Let *n* be the cells density defined e.g. as the number of circles’ centers in a unit area. According to [31, 32], emergence of large groups of interconnected cells, the percolation transition, in a set of randomly distributed circles can be characterised by the mean number of centers that fall within a circle of radius *R*:

$$\begin{array}{c} B=\pi R^{2}n. \end{array}$$ (1)

In particular, there exists a critical concentration *B* = *B*_*c*_ at which two arbitrary circles become connected with high probability. Thus percolation occurs and a large cluster of connected circles appears. In contrast with typical thermal phase transitions, where a transition between two phases occurs at a critical temperature, percolation transition relates to distribution and topology of clusters corresponding to the values of *B* in a neighborhood of *B*_*c*_. At low values of *B* only small clusters of overlapping circles exist. When the concentration *B* increases the average size of the clusters increases too. At the critical concentration value, *B* = *B*_*c*_, a large cluster appears; it includes groups of cells that are close to the opposing boundaries of the original square. This cluster is called *spanning cluster* or percolating cluster. In the thermodynamic limit, i.e. in the infinite size system limit the spanning cluster is called *infinite cluster*. For scalar problem the value of *B*_*c*_ ≈ 1.1.

*Case 2*. Cells have axons, *H* > 0, and axons are allowed to transmit signals in both directions. Each neuron can be represented as an undirected pair of head- and tail-circles both having radius *R*. When the head-circle or the tail-circle of an neuron overlaps with the head- or the tail-circles of another neuron we consider these neurons connected. Despite this setting differs from Case 1 in that we now operate with dipoles rather than with just circles, the problem remains within a class of scalar percolation, albeit for dipoles of circles not just a single circle.

*Case 3*. Cells have axons, *H* > 0, and these axons can only transmit signals along a straight line which determines direction of connectivity for a given cell. The coupling direction from soma to synaptic terminal has isotropic distribution, and hence each neuron could be represented as a directed pair of head- and tail-circles both having radius *R*. Vectors linking centers of the head- and tail-circles are allowed to have arbitrary direction. Their lengths, *H*, however, are fixed. When the tail-circle of a neuron overlaps with a head-circle of another neuron the pair is considered as connected. In contrast with two other ways of establishing neuronal connectivity considered above this is the most realistic scenario. It is no longer within the scope of simple scalar circle percolation framework but is a vector percolation problem.

**Fig 1:**
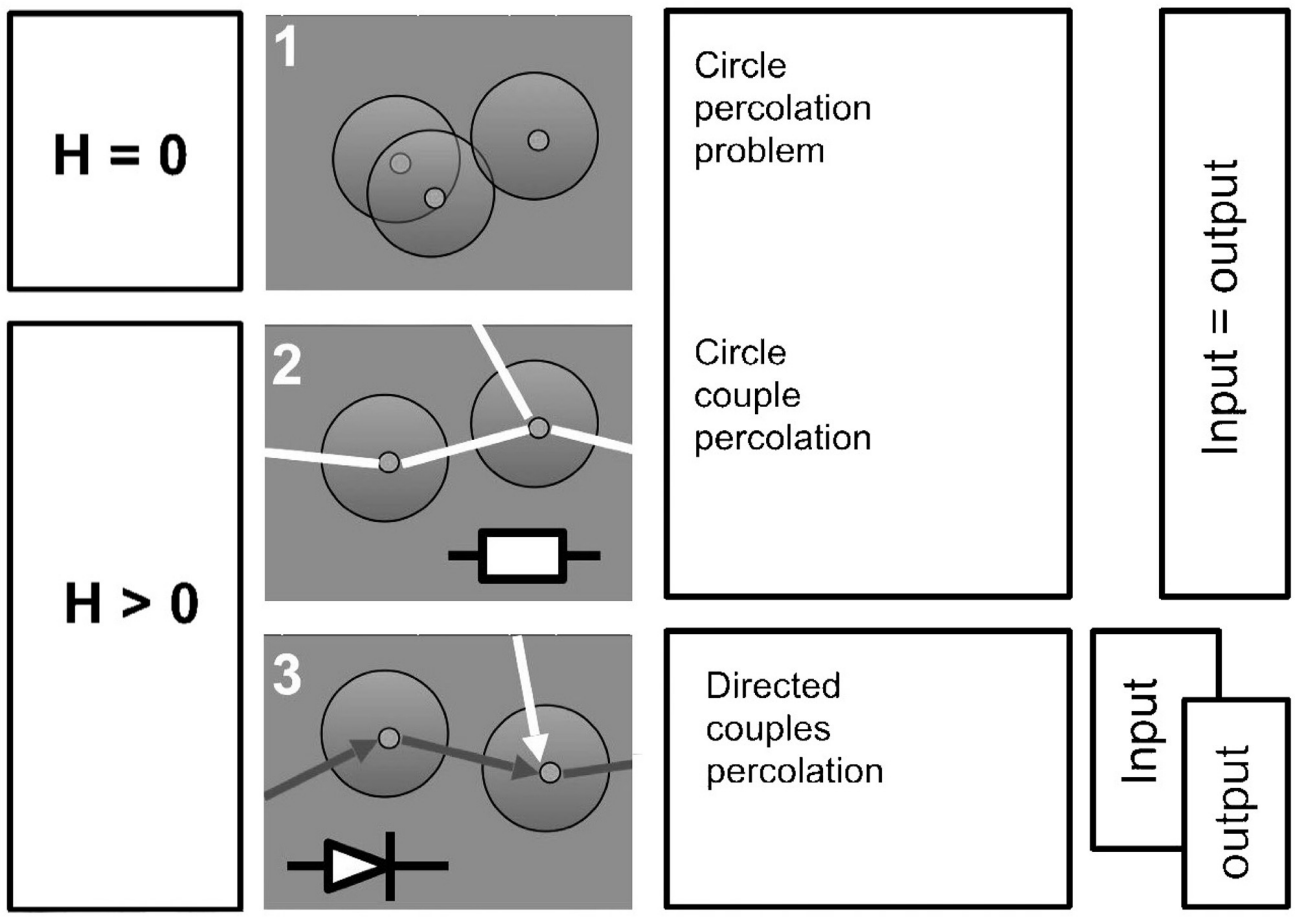
Schematic representation of three different percolation settings for the geometrical model.

**Fig 2:**
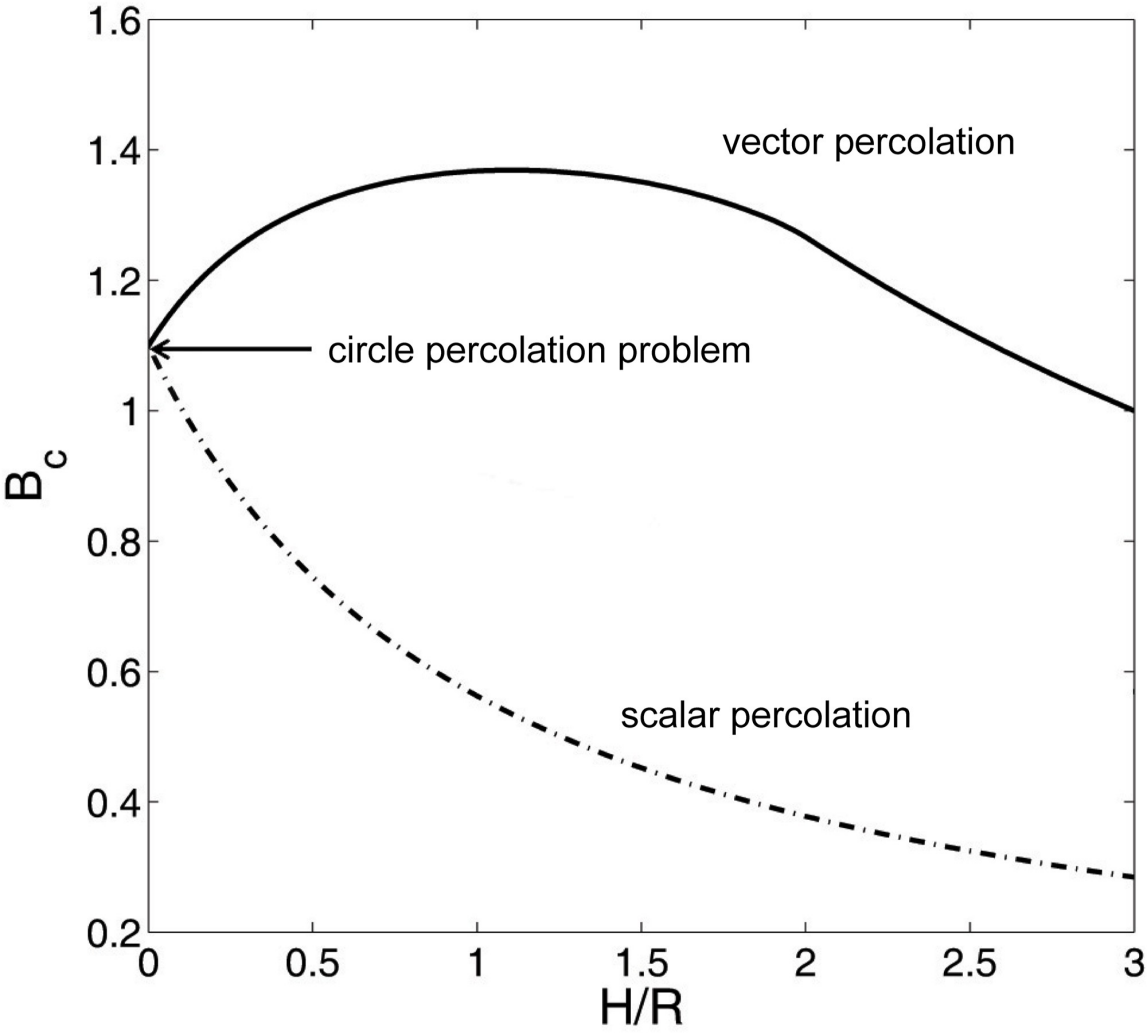
Dependence of the percolation threshold parameter, *B*_*c*_, on *H*/*R* for 2D scalar and vector percolation problems (Case 2 and 3 respectively).

The three cases are illustrated with Fig 1. Fig 2 shows dependence of the percolation threshold parameter, *B*_*c*_, on the ratio *H*/*R*. In accordance with he definition of *B* in (1) the cell’s concentration variable *B* can be related to the expected number of neurons that are connected to a randomly chosen query neuron in the system. The latter quantity is known as the average network coordination number *z*. As can be seen from Fig 2 the value of the coordination number *z* that corresponds to *B*_*c*_ is always bounded from above. *B*_*c*_ ≤ 1.4 for all configurations considered so far.

The above analysis reveals that networks with coordination numbers exceeding these critical values are likely to form a spanning cluster that is capable of connecting opposite edges of the system [33, 34]. Thus, intuitively, one can argue that signals initiated by spontaneous activation of neurons in the cluster can spark waves of activity through the whole network. Such conclusion is based on the restrictive assumption that neurons always elicit spikes in response to a spike on their input. Not only this assumption does not necessarily hold true, but also the above geometrical model alone does not explain the wealth of excitation propagation phenomena observed in cultures. To account for more plausible situations, as well as to possibly increase explanatory power of the model, two additional variables are introduced: one is the probability *p* of neuronal activation in response to incoming spike, and the other is an exogenous “resource” variable *E* determining if a neuron has enough energy to elicit a spike. The consequences of adding these two variables are discussed in the next sections.

#### Mean-field dynamic model of neuronal excitation

We begin with a simple mean-field approximation of the dynamics of neural excitation in the system. Consider a connected network of neuronal cells. Let *z* be the expected coordination number, i.e. the expected number of neighbors of a randomly chosen query cell. Suppose that at a time instance *t* some neurons in the network are excited. Let *q*_*t*_ denote the number of these neurons relative to the total number of cells the whole network. If the value of *z* is sufficiently large then the number of excited neurons among all neighbors of a given neuron can be estimated as *zq*_*t*_. Let *p* be the probability of neuronal activation in response to the activation of at least one neuron from its nearest neighbours, and suppose that all excitatory signals are independent. Thus the probability that a given neuron is activated equals to $1-(1-p{)}^{zq_{t}}$, and hence the expected proportion of all excited neurons at the time step *t* + 1 is:

$$\begin{array}{c} q_{t+1}=1-{\left(1-p\right)}^{zq_{t}}. \end{array}$$ (2)

The range of dynamics which model (2) is capable to reproduce is summarized in Proposition 1.

**Proposition 1** *Consider* (2) *and let p* ∈ (0, 1), *z* > 0 *be constants. Then the interval* [0, 1] *is forward-invariant, all forward orbits q*_*t*_ *are monotone functions t*, *and the point map* (2) *has only fixed points as attractors. Furthermore*

1. *if* − *z* log(1 − *p*) ≤ 1 *then the map has only one fixed point*, $q_{1}^{*}=0$, *and it is an attractor*;
2. *if* −*z* log(1 − *p*) > 1 *then the map has only two fixed points with* $q_{1}^{*}=0$ *being a repeller and the other one*, $q_{2}^{*}\in (0,1)$, *a stable attractor*.

Proof of Proposition 1 is provided in the Appendix.

According to Proposition 1, asymptotic mean-field dynamics (3) of the network is a steady activity at an equilibrium in the entire domain of the model’s feasible parameters: *p* ∈ (0, 1), *z* > 0. Moreover, all transients are monotone trajectories. Emergence of the unique non-zero asymptotic activity is fully determined by the values of the connectivity parameter, *z*, and the probability of neural activation, *p*. Critical values of these parameters, e.g. the critical connectivity, ${\bar{z}}_{1}$, at which the transition occurs, satisfies:

$$\begin{array}{c} {\bar{z}}_{1}=\frac{-1}{\log(1-p)}. \end{array}$$ (3)

For ${\bar{z}}_{1}\gg 1$, this relation is approximately reciprocal: $p≃1/{\bar{z}}_{1}$. Indeed, expressing $p=1-\text{exp}\left(-\frac{1}{{\bar{z}}_{1}}\right)$ from (3), and expanding $\text{exp}\left(-\frac{1}{{\bar{z}}_{1}}\right)$ as a power series with respect to $1/{\bar{z}}_{1}$, one obtains:

$$\begin{array}{c} p=\frac{1}{{\bar{z}}_{1}}+O(\frac{1}{{{\bar{z}}_{1}}^{2}}). \end{array}$$

The value of the stable equilibrium, $q_{2}^{*}$, can be estimated as follows. At the steady state we have that $1-q_{2}^{*}=(1-p{)}^{zq_{2}^{*}}$. Hence, $(1-q_{2}^{*}{)}^{1/q_{2}^{*}}=(1-p{)}^{z}$. It is well known that (1 − *x*)*e*^−1^ < (1 − *x*)^1/*x*^ for all *x* ∈ (0, 1). Thus

$$\begin{array}{c} \left(1-q_{2}^{*}\right)<e{\left(1-p\right)}^{z}\Rightarrow q_{2}^{*}>1-e{\left(1-p\right)}^{z}. \end{array}$$

Observe that the larger the value of the connectivity parameter, *z*, is, the higher is the level of the mean-field network activity.

Whilst model (3) is consistent with the very basic observation that increasing the network’s overall connectivity may lead to emergence of a self-sustained activity in the network, the model’s explanatory capability is limited. The model does not explain widely-reported richness of the dynamics in live neuronal cultures, including emergence of spontaneous activity bursts and irregular and seemingly chaotic spikes.

This limitation is not surprising since (2) is a crude approximation of the network’s dynamics. Model (2) does not account for a broad spectrum of biological mechanisms involved in spike generation and assumes that the neuron’s ability to produce spikes depends exclusively on stimulation. A possible way to overcome this unrealistic assumption is to explicitly account for these missing mechanisms. To keep the model simple, we account for joint effect of these mechanisms by adding a single energy-like variable *E*_*t*_ to (2). The new variable determines the neuron/network’s ability to produce a spike depending on the amount of resources or “energy” available. Generic models of this type have been proposed and analyzed in [27, 35] in the context of adaption to stress and external environmental factors. These models have been shown to capture periodic and irregular behavior in multi-agent systems [28].

**Fig 3:**
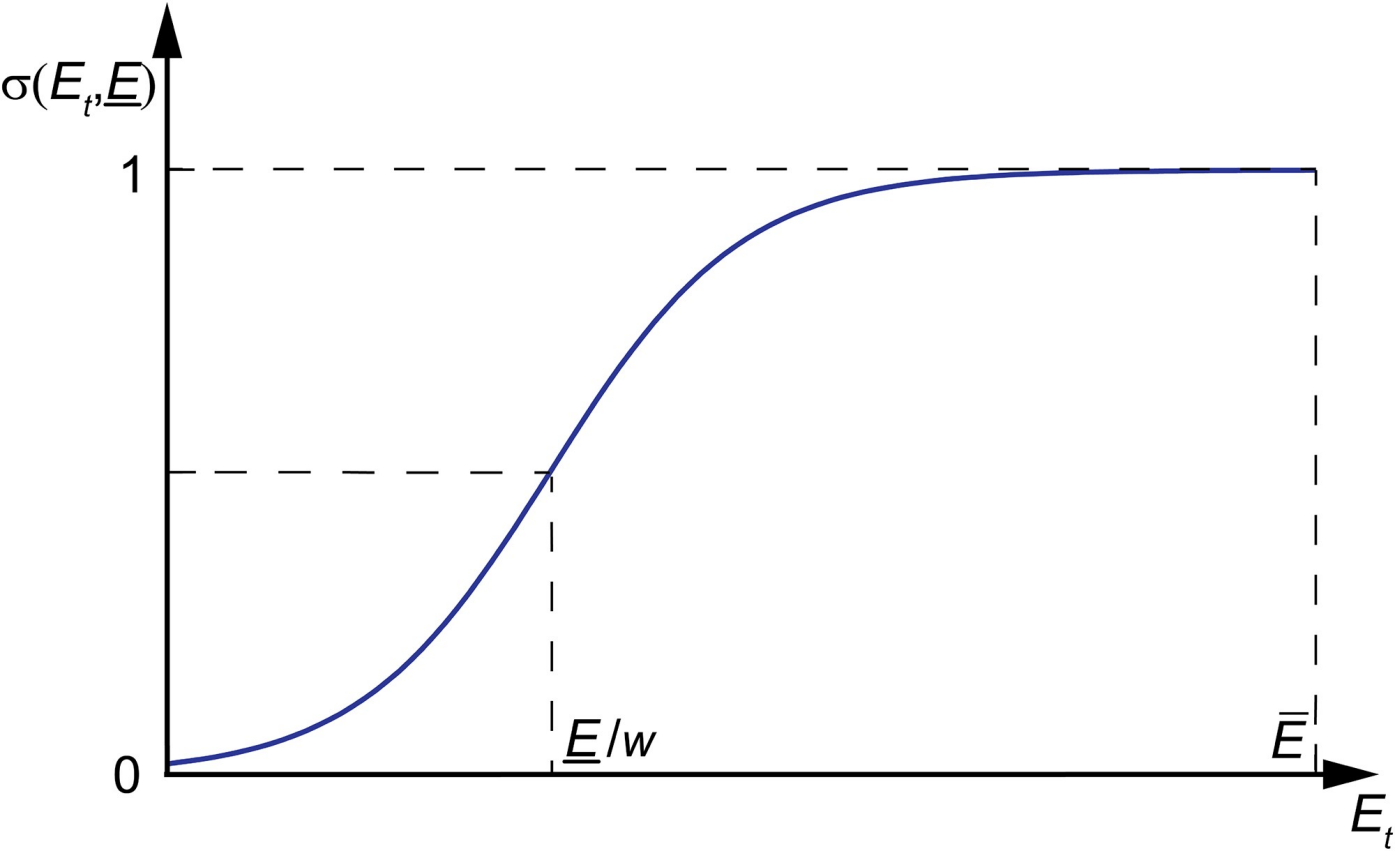
General shape of the function $\sigma (\cdot ,\underline{E})$.

Here we extend the original phenomenological mean-field model (2) as follows:

$$\begin{array}{c} \begin{array}{cc} q_{t+1}= & 1-{\left(1-p\cdot \sigma \left(E_{t},\underline{E}\right)\right)}^{zq_{t}}\\ E_{t+1}= & \left(1-\varepsilon \right)E_{t}+\varepsilon \bar{E}-rq_{t}H(E_{t}-rq_{t}), \end{array} \end{array}$$ (4)

where

$$\begin{array}{c} \sigma \left(E_{t},\underline{E}\right)=\frac{1}{2}\left(\tanh\left(wE_{t}-\underline{E}\right)+1\right), \end{array}$$

and H is the Heaviside function. In (4) *p* is the maximal probability of neuronal activation, *z* > 0 is the coordination number, *E*_*t*_ is the exogenous phenomenological “energy resource” variable; *r* > 0 is the energy cost of neuronal activation, $\underline{E}>0$ and *w* > 0 are parameters that determine the minimal activation probability and the energy activation threshold, $\bar{E}>\underline{E}$ is the energy recovery value, *ε* ∈ (0, 1) is the energy relaxation parameter. The general shape of the function $\sigma (\cdot ,\underline{E})$ in the energy-dependent synaptic efficacy component, $p\sigma (\cdot ,\underline{E})$, is shown in Fig 3.

Mean-field model (4) of the network dynamics inherits phenomenological transparency of (3). It does, however, account for generic constraints of spike-generation through the new energy variable *E*_*t*_ and energy-modulated synaptic efficacy $p\sigma (\cdot ,\underline{E})$. Despite retaining simplicity, the model produces remarkably rich dynamics. Its equilibria, however, can still be described by just a few parameters as follows from Proposition 2 below.

**Proposition 2** *Consider* (4) *with p* ∈ (0, 1). *Then the domain*

$$\begin{array}{c} \left\{\left(q,E\right)\;|q\in \left[0,1\right],E\in \left[0,\bar{E}\right]\right\} \end{array}$$

*is forward-invariant. In addition*,

1. *If* $-z\;\text{log}\left(1-p\sigma \left(\bar{E},\underline{E}\right)\right)\leq 1$ *then* (4) *has only one fixed point*:
  $$\begin{array}{c} \left(q_{1}^{*},E_{1}^{*}\right)=\left(0,\bar{E}\right). \end{array}$$ (5)
  *This fixed point is an attractor when* $-z\;\text{log}\left(1-p\sigma \left(\bar{E},\underline{E}\right)\right)<1$.
2. *If* $-z\;\text{log}\left(1-p\sigma \left(\bar{E},\underline{E}\right)\right)>1$ and $\bar{E}/r\geq 1$ *then* (4) *has at most two fixed points: fixed point* (5) *and, if exists, an additional fixed point* $\left(q_{2}^{*},E_{2}^{*}\right)$:
  $$\begin{array}{c} \begin{array}{cc} & \left(q_{2}^{*},E_{2}^{*}\right),\;0<q_{2}^{*}\leq \frac{\bar{E}}{r(1+\frac{1}{\varepsilon })},\;q_{2}^{*}<1,\\ & q_{2}^{*}=1-{\left(1-p\sigma (\bar{E}-\frac{rq_{2}^{*}}{\varepsilon },\underline{E})\right)}^{q_{2}^{*}z},\;E_{2}^{*}=\bar{E}-\frac{rq_{2}^{*}}{\varepsilon }. \end{array} \end{array}$$ (6)
  *The fixed point* $\left(q_{1}^{*},E_{1}^{*}\right)$ *is a repeller*.
3. *If* $-z\;\text{log}\left(1-p\sigma \left(\bar{E},\underline{E}\right)\right)>1$ and $\bar{E}/r<1$ *then* (4) *has at most three fixed points. The fixed point* $\left(q_{1}^{*},E_{1}^{*}\right)$ *specified by* (5) *and, possibly, additional two fixed points: the fixed point* $\left(q_{2}^{*},E_{2}^{*}\right)$ *specified by* (6) *and a fixed point*
  $$\begin{array}{c} \begin{array}{cc} & \left(q_{3}^{*},E_{3}^{*}\right),\;1>q_{3}^{*}>\frac{\bar{E}}{r},\\ & q_{3}^{*}=1-{\left(1-p\sigma \left(\bar{E},\underline{E}\right)\right)}^{q_{3}^{*}z},\;E_{3}^{*}=\bar{E}. \end{array} \end{array}$$ (7)
  *The fixed point* $\left(q_{1}^{*},E_{1}^{*}\right)$ *is a repeller, and* $\left(q_{3}^{*},E_{3}^{*}\right)$, *if exists, is a stable attractor*.

Proof of Proposition 2 is provided in the Appendix.

**Fig 4:**
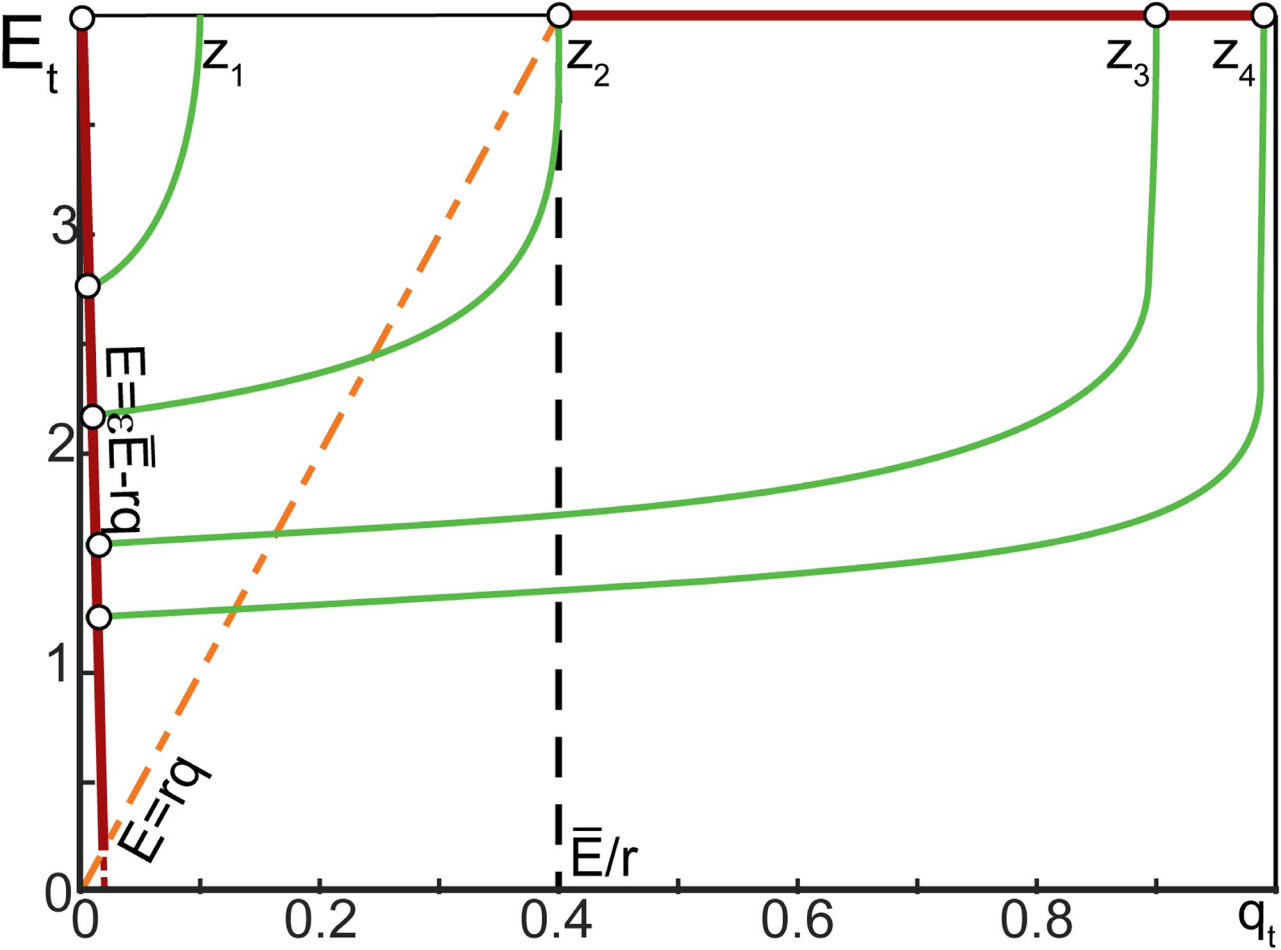
Arrangement of equilibria in (4). The model parameters were set as follows: *w* = 1.5, *p* = 0.1, $\bar{E}=4$, $\underline{E}=2$, *ε* = 0.05, *r* = 10, with *z* taking values in the set {10, 12.2, 25, 50} (these values are denoted as *z*_1_, *z*_2_, *z*_3_ and *z*_4_).

An illustration showing relationships between parameters of the model and emergence of the three different equilibria described in Proposition 2 is provided in Fig 4. The equilibria are shown as white circles. Green lines show curves

$$\begin{array}{c} E^{*}=\sigma^{-1}\left(q^{*},\underline{E}\right)=\frac{1}{w}\tanh^{-1}(\frac{2}{p}(1-{\left(1-q^{*}\right)}^{\frac{1}{q^{*}z}})-1)+\frac{\underline{E}}{w} \end{array}$$ (8)

as functions of q* > 0. According to the first equation of (4), all equilibria of the model with q* ≠ 0 must belong to these curves (see also the proof of Proposition 2 in Appendix). Depending on the value of *z*, the curves move up and down, and intersect with line segments (shown as red solid lines in Fig 4):

$$\begin{array}{c} E^{*}=\bar{E}-r\frac{q^{*}}{\varepsilon },\;0<q^{*}\leq \frac{\bar{E}}{r(1+\frac{1}{\varepsilon })},\;q^{*}<1 \end{array}$$

and

$$\begin{array}{c} E^{*}=\bar{E},\;\frac{\bar{E}}{r}<q^{*}<1. \end{array}$$

These intersections correspond to equilibria (6) and (7), respectively. The equilibria persist over intervals of *z*, and the greatest lower bounds of these intervals (critical values of *z*) are:

$$\begin{array}{c} {\bar{z}}_{2}=\frac{-1}{\log(1-p\sigma \left(\bar{E},\underline{E}\right))}\;\text{and}\;\;{\bar{z}}_{3}=\frac{\log(1-\frac{\bar{E}}{r})}{\frac{\bar{E}}{r}\log\left(1-p\sigma \left(\bar{E},\underline{E}\right)\right)},\;\frac{\bar{E}}{r}<1. \end{array}$$ (9)

We also note (see the proof of Proposition 2) that equilibria (6) are always above or on the line *E* = *rq* (dashed orange line in Fig 4), whereas equilibria (7) are to be below this line.

According to Propositions 1, 2, models (2) and (4) share some similarity. For *z* sufficiently small, all orbits are attracted to a single equilibrium. At this equilibrium, the systems are silent. When *z* increases and eventually exceeds the first critical value (Eq (3) for (2) and ${\bar{z}}_{2}$ for (4)), the silent equilibrium becomes a repeller and the systems start to exhibit non-zero activity. However, further increases of *z* trigger drastically different dynamics in these models.

**Fig 5:**
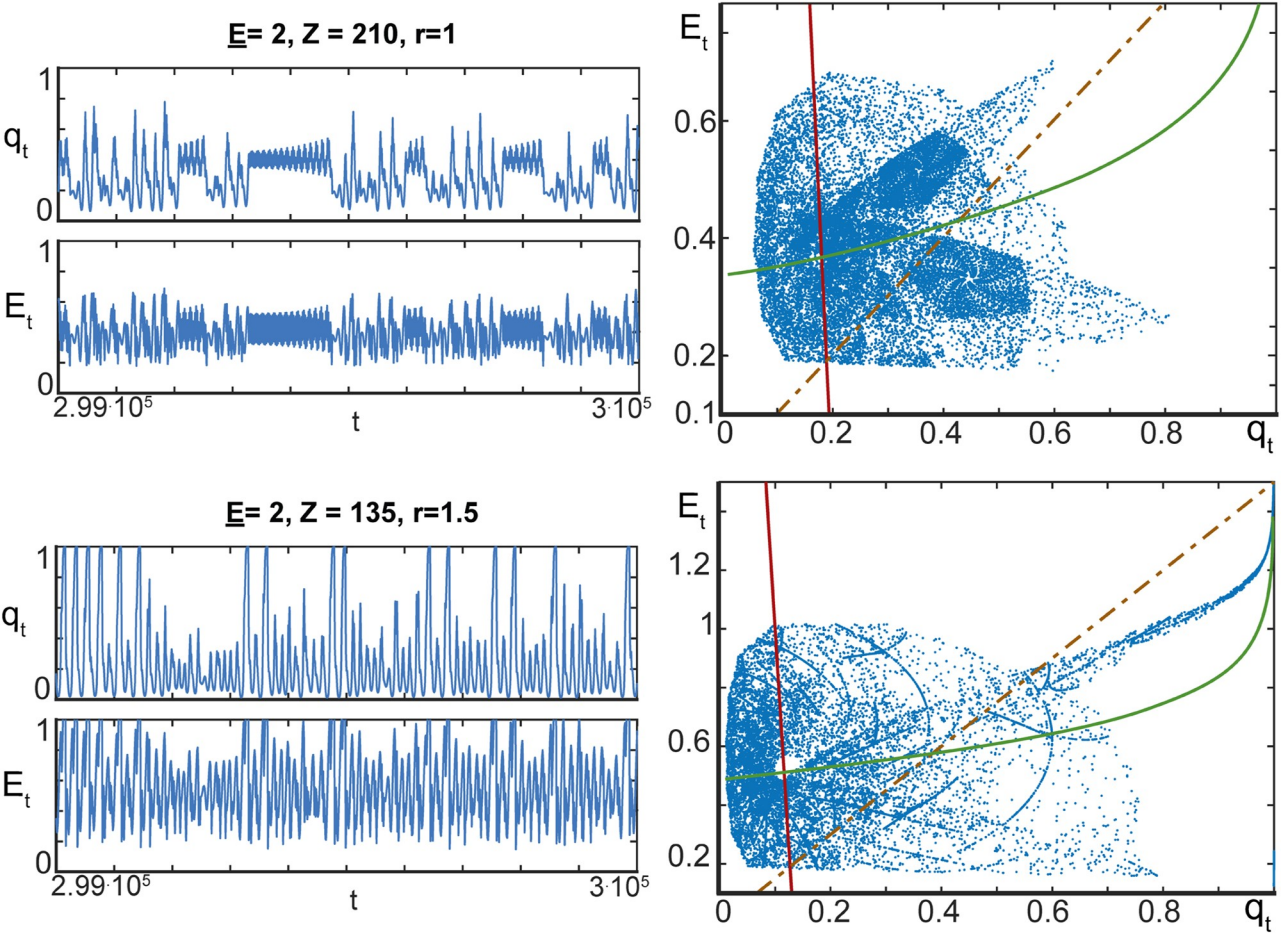
Complex behaviour of orbits generated by (4). *Top panels* correspond to *z* = 210, *r* = 1, and *bottom panels* show dynamics of (4) for *z* = 135, *r* = 1.5. Other parameters of the model are: *w* = 1.5, *p* = 0.1, $\bar{E}=4$, $\underline{E}=2$, *ε* = 0.05. Green curves show (8), solid red line shows $E^{*}=\bar{E}-rq^{*}/\varepsilon$, and dashed orange line corresponds to E* = rq*. Intersection of the solid green and red curves above the dashed curve reveals equilibrium (6). *Left panels* present the evolution of *q*_*t*_ and *E*_*t*_ as functions of *t*. *Right panels* show the values of pairs (*q*_*t*_, *E*_*t*_) for *t* ∈ [2.8 ⋅ 10^5^, 3 ⋅ 10^5^].

All orbits of model (2) with *q*_0_ ≠ 0, as ensured by Proposition 1, converge monotonically to a single non-zero steady state regardless of how large the values of *z* become. The spectrum of orbits in model (4) is different. Our numerical experiments demonstrated that, in addition to equilibria, the model is capable of generating periodic orbits too. Moreover, for a broad range of parameters it produced complicated and apparently chaotic motions. Examples of these complicated motions are shown in Fig 5. Observe that the model parameters corresponding to the trajectories in Fig 5 satisfy statement 2) of Proposition 2. In this case, at most two equilibria may exist. As we can see from Fig 5, these equilibria (fixed points (5) and (6)) are not attracting the orbits, and trajectories appear to be chaotic with some apparent intermittency.

**Fig 6:**
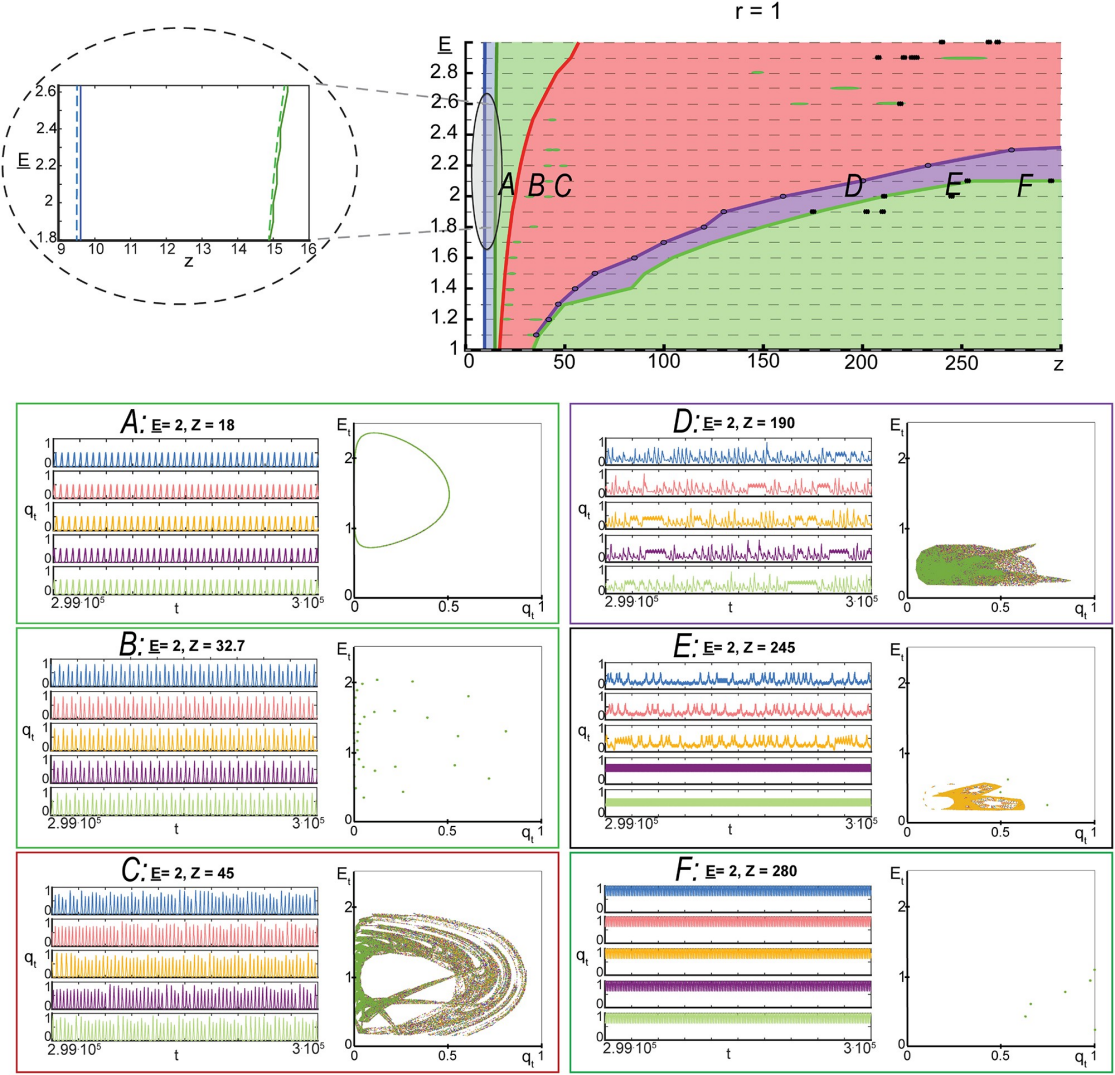
Complex dynamics of model (4). *Top row*: parametric portrait of the qualitative dynamics of model (4) in the domain $\left\{(\underline{E},z)|\underline{E}\in [1,3],\;z\in (0,300]\right\}$ at *r* = 1, *w* = 1.5, *p* = 0.1, $\bar{E}=4$, *ε* = 0.05. White areas show the parameter regions in which only one fixed point, (5), was detected. This fixed point is an attractor. The blue region bordering the white one corresponds to the case in which fixed point (5) becomes a repeller and the second equilibrium, (6), emerges. Equilibrium (6) is locally asymptotically stable. Green areas are the domains in which an attracting periodic orbit was detected. Red and violet domains correspond to regions where complex chaotic-like dynamics were observed. Black stars, *, indicate observed co-existence of multiple attractors. Inlet linked to the gray area shows theoretical estimates of transition boundaries (dashed blue and green lines) relative to the ones observed in experiments. *A,B,C,D,E,F*: typical dynamics observed in the corresponding parametric regions.

**Fig 7:**
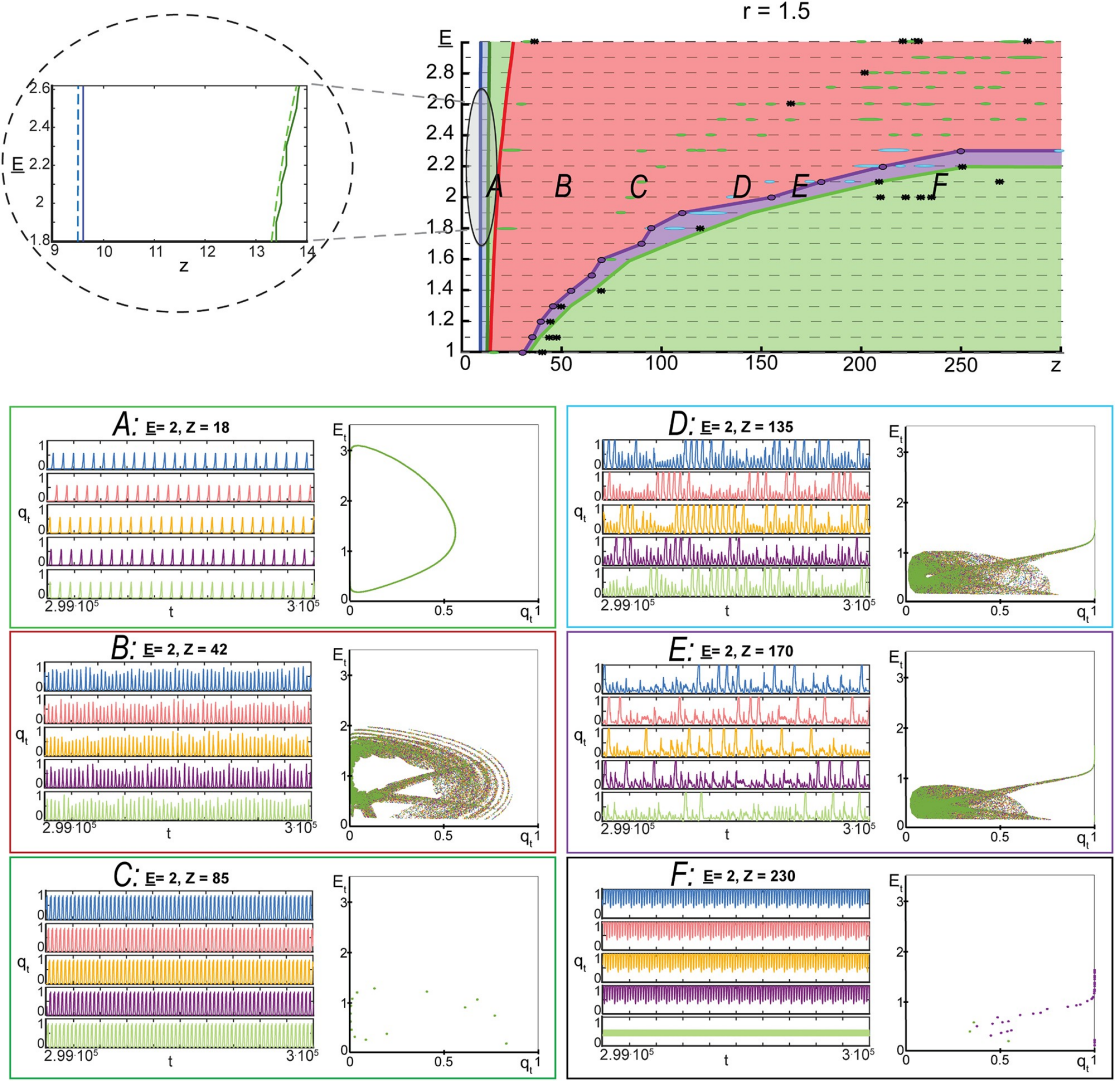
Complex dynamics of model (4). *Top row*: parametric portrait of the qualitative dynamics of model (4) in the domain $\left\{(\underline{E},z)|\underline{E}\in [1,3],\;z\in (0,300]\right\}$ at *r* = 1.5, *w* = 1.5, *p* = 0.1, $\bar{E}=4$, *ε* = 0.05. White areas show the parameter regions in which only one fixed point, (5), was detected. This fixed point is an attractor. The blue region bordering the white one corresponds to the case in which fixed point (5) becomes a repeller and the second equilibrium, (6), emerges. Equilibrium (6) is locally asymptotically stable. Green areas are the domains in which an attracting periodic orbit was detected. Red and violet domains correspond to regions where complex chaotic-like dynamics were observed. Black stars, *, indicate observed co-existence of multiple attractors. Turquoise blue islands mark regions in which burst-like trajectories were observed. Inlet linked to the gray area shows theoretical estimates of transition boundaries (dashed blue and green lines) relative to the ones observed in experiments. *A,B,C,D,E,F*: typical dynamics observed in the corresponding parametric regions.

**Fig 8:**
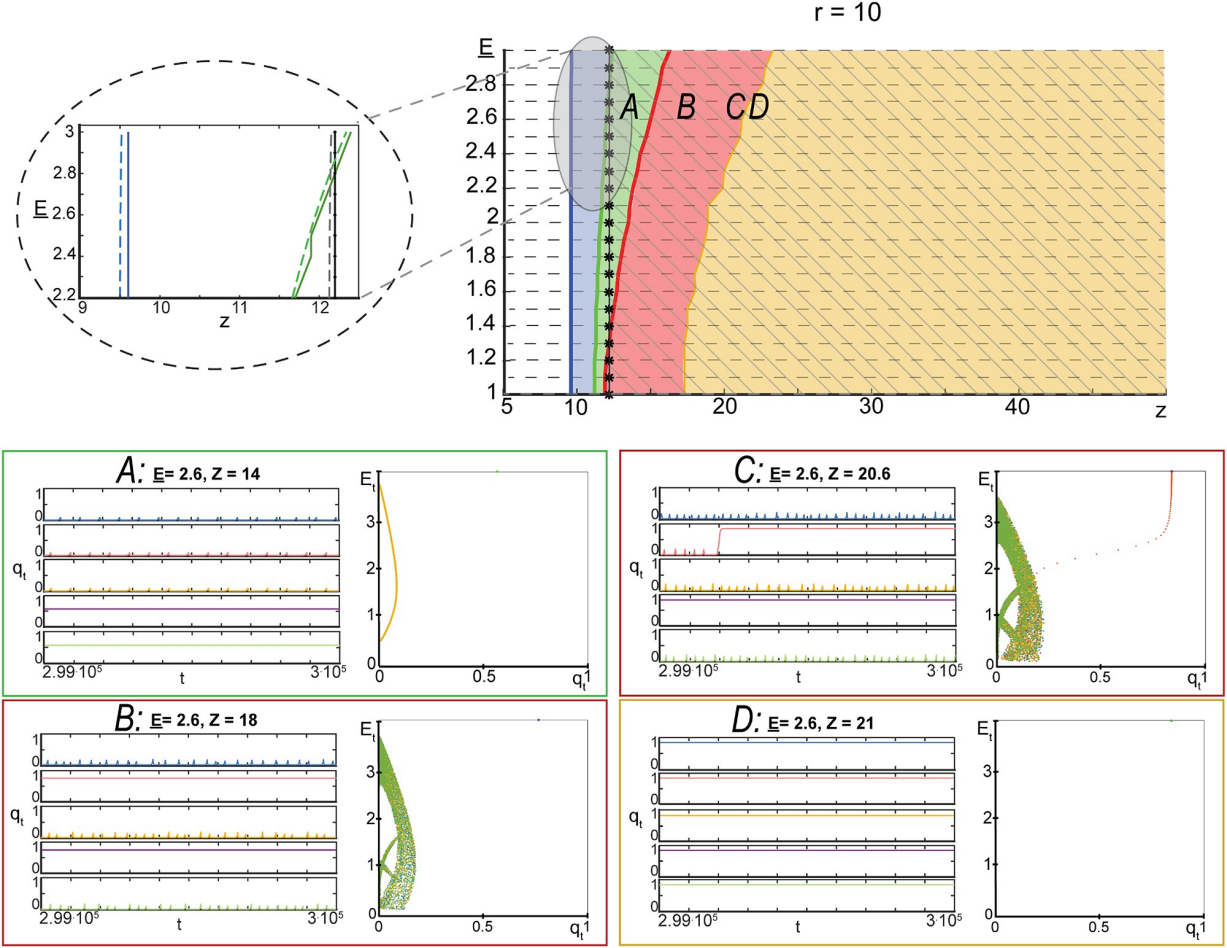
Complex dynamics of model (4). *Top row*: parametric portrait of the qualitative dynamics of model (4) in the domain $\left\{(\underline{E},z)|\underline{E}\in [1,3],\;z\in (0,50]\right\}$ at *r* = 10, *w* = 1.5, *p* = 0.1, $\bar{E}=4$, *ε* = 0.05. White areas show the parameter regions in which only one fixed point, (5), was detected. This fixed point is an attractor. The blue region bordering the white one corresponds to the case in which fixed point (5) becomes a repeller and the second equilibrium, (6), emerges. Equilibrium (6) is locally asymptotically stable. Green areas are the domains in which an attracting periodic orbit was detected. The red area corresponds to regions where complex chaotic-like dynamics were observed. The black line indicates a boundary beyond which co-existence of multiple attractors was observed consistently in all experiments. Inlet linked to the gray area shows estimates of transition boundaries (dashed blue (${\bar{z}}_{2}$ in (9)), green, and black (${\bar{z}}_{3}$ in (9)) lines) relative to the ones observed in experiments. The yellow region shows the domain in which all trajectories converged to (7). *A,B,C,D*: typical dynamics observed in the corresponding parametric regions.

In order to gain additional insight into the model’s dynamics, we numerically explored asymptotic regimes of (4) for varying values of *z*, $\underline{E}$, and *r*. Other parameters were as follows: *ε* = 0.05, *w* = 1.5, $\bar{E}=4$, *p* = 0.1. Outcomes of these experiments are summarized in Figs 6–8 (see Materials and methods for details of the steps taken to produce these figures). In these experiments, the values of $\underline{E}$ were chosen from a uniform equispaced grid of 21 points in the interval [1, 3]. This grid is shown as grey dashed horizontal lines in Figs 6–8. Parameter *z* was varying adaptively (increments ranged from 0.1 in the intervals (0, 50] and (200, 300] to 5 in the interval (50, 200]). For these values of model parameters, we assessed the type of the model’s asymptotic dynamics and mapped these onto relevant parametric regions. These regions are shown with different colour in Figs 6–8.

According to Figs 6–8, development and evolution of the dynamics of (4) follows a robust pattern. For a fixed value of $\underline{E}\in [1,3]$ and *z* small, trajectories of the model converge to a unique attractor (fixed point 5). This attractor corresponds to the system’s state in which no elements/neurons are excited. When the value of *z* increases and exceeds ${\bar{z}}_{2}$ (specified in (9) and shown as blue dashed lines in 6–8), equilibrium (5) becomes a repeller and a second equilibrium emerges (fixed point (6). Numerical evaluation of the eigenvalues of the Jacobian at this equilibrium showed that it is locally asymptotically stable. Further increments of *z* lead to that fixed point (6) loses stability thorough the Neimark-Sacker bifurcation, and an attracting periodic orbit emerges. The boundary of this transition is depicted as green dashed lines in Figs 6–8. As we keep increasing the values of *z*, non-trivial and complex dynamics eventually occur (red area in Figs 6–8). Complex orbits and behavior persist over intervals of values of *z*.

Emergence of rich activity patterns in neuronal as a direct result of the network connectivity has been investigated in [36]. It has been found in [36] that the generation and spatiotemporal patterns of propagation were most variable in networks with intermediate clustering and lowest in uniform networks. Whilst our model (4) does not allow to discriminate between clustered and uniform connectivity explicitly, Figs 6–8 capture the general connectivity effect on the system’s dynamics: rich and complex trajectories occur at the “intermediate” values of connectivity parameter *z* (see e.g. Fig 8). Figs 6–8 also show that exact bounds of such “intermediate” values of *z* may depend on the values of other model parameters.

Notice that some of the complex trajectories shown in panel *C*, 8 eventually converge to the stable equilibrium specified by (7). This is an empirical manifestation of slow relaxations and critical delays in model (4) [37], [38]. For *z* sufficiently large, these complex orbits disappear and reduce to periodic orbits, Figs 6 and 7, or mere equilibria, Fig 8.

Mean-field bursting dynamics, shown e.g. in panels D and E in Figs 6 and 7, resembles that of the population bursts observed in live neuronal evolving cultures. An important factor in successful replication of this behavior was the energy variable, *E*_*t*_, coupled with the energy-dependent activation probability $p\sigma (E_{t},\underline{E})$. The mean-field model, however, does not capture spatial effects and as such is only a rough approximation of activity propagation in neuronal cultures. In the next section we extend the proposed mean-filed model (4) to a multi-agent network with randomized energy-dependent activation and numerically assess relevant parameters of its dynamics, including distributions of sizes and durations of firing avalanches.

#### Multi-agent model of neuronal excitation

As a natural extension of (4), we consider a connected network of *N* neurons. The network’s topology is determined by an adjacency matrix, *C*, whose elements *c*_*ij*_ are:

$$\begin{array}{c} c_{ij}=\{\begin{array}{cc} 1, & \text{if}\;\text{there}\;\text{is}\;\text{a}\;\text{link}\;\text{from}\;\text{the}\;i\text{-th}\;\text{node}\;\text{to}\;\text{the}\;j\text{-th}\\ 0, & \text{otherwise}. \end{array} \end{array}$$

No links from a node to itself are permitted, but cycles are allowed. For simplicity, all links in the network have been assigned equal weights of which the value was assumed to be 1. For the given adjacency matrix *C*, we determined the average number of inputs, 〈*N*_in_〉, and the average number of outputs 〈*N*_out_〉

$$\begin{array}{c} \left\langle N_{\text{in}}\right\rangle =\frac{1}{N}\sum_{i=1}^{N}\sum_{j=1}^{N}c_{ji},\;\left\langle N_{\text{out}}\right\rangle =\frac{1}{N}\sum_{i=1}^{N}\sum_{j=1}^{N}c_{ij}. \end{array}$$

Dynamics of each *i*-th node in the network is described by two variables: the activity variable, $q_{i,t}\in \mathbb{R}$, and the energy variable, $E_{i,t}\in \mathbb{R}$. Equations governing evolution of these variables have been defined as follows:

$$\begin{array}{c} \begin{array}{cc} q_{i,t+1} & =a_{i,t};\\ E_{i,t+1} & =\left(1-\varepsilon \right)E_{i,t}+\varepsilon \bar{E}-a_{i,t}E_{i,t}^{\text{fire}}, \end{array} \end{array}$$ (10)

where

$$\begin{array}{c} E_{i,t}^{\text{fire}}=\frac{r_{1}\sum_{j=1}^{N}c_{ij}}{\left\langle N_{\text{out}}\right\rangle }+\frac{r_{2}\sum_{j=1}^{N}c_{ji}q_{j,t}}{\left\langle N_{\text{in}}\right\rangle }, \end{array}$$

and

$$\begin{array}{c} \begin{array}{cc} a_{i,t} & =\{\begin{array}{cc} 1\;\text{with}\;\text{probability}\;\;p_{i,t}^{\text{fire}}, & \text{if}\;E_{i,t}>E_{i,t}^{\text{fire}};\\ 0, & \text{otherwise}, \end{array}\\ p_{i,t}^{\text{fire}} & ={\left(1-p\sigma \left(E_{i,t},\underline{E}\right)\right)}^{1+\sum_{j=1}^{n}c_{ji}q_{j,t}}. \end{array} \end{array}$$

The variables *q*_*i*,*t*_ take values in the set {0, 1}, and *E*_*i*,*t*_ are in the interval $\left[0,\bar{E}\right]$. The function $\sigma (\cdot ,\underline{E})$ is as in (4).

Phenomenological motivation for the dynamics of individual nodes in model (10) is similar to that of the mean-field model, (4). There are, however, several key differences. The evolution of variables *q*_*i*,*t*_ and *E*_*i*,*t*_ in (10) explicitly accounts for local network topology and is directly driven by activity of the node’s neighboring cells (as opposed to mean connectivity *z* and activity in (4)). The energy balance equation, the second equation in (10), accounts for the costs of transmitting active signals at the neuron’s input (term $r_{2}\sum_{j=1}^{N}c_{ji}q_{j,t}{\langle N_{in}\rangle }^{-1}$) and generating activity signals on the neuron’s output (term $r_{1}\sum_{j=1}^{N}c_{ij}{\langle N_{out}\rangle }^{-1}$). If the node’s energy level is insufficient to trigger a spike, $E_{i,t}\leq E_{i,t}^{fire}$, then no spikes are generated at *t*+ 1. The latter property is difficult to fully capture at the level of the mean-field approximation, as low sub-threshold values of the bulk energy do not necessarily imply absence of activity at the level of individual neurons (cf. Proposition 2, alternative 3, and Fig 4).

In our numerical experiments, we focused largely on fully connected networks for which *c*_*ij*_ = 1 − *δ*_*ij*_, where *δ*_*ij*_ is the Kronecker’s delta. Addition of a fraction of inhibitory connections did not result in qualitative changes in the network’s dynamics. These simplifications are consistent with the approaches and observations reported in earlier works [23]. The model parameters where set as follows:

$$\begin{array}{c} p=0.01,\;\underline{E}=2,\;\bar{E}=4,\;w=1.5,\;\varepsilon =0.0025,\;N=625, \end{array}$$

and parameters *r*_1_ and *r*_2_ varied in the intervals [1, 1.5] and [4, 6], respectively.

**Fig 9:**
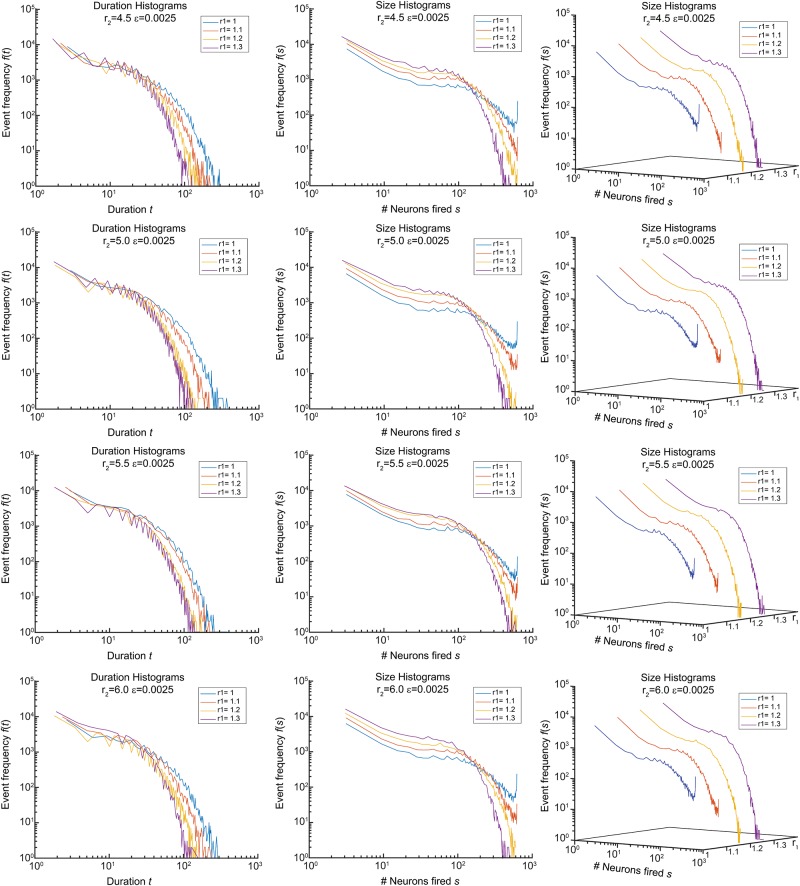
Sizes and durations of avalanches for different parameters of the model.

**Fig 10:**
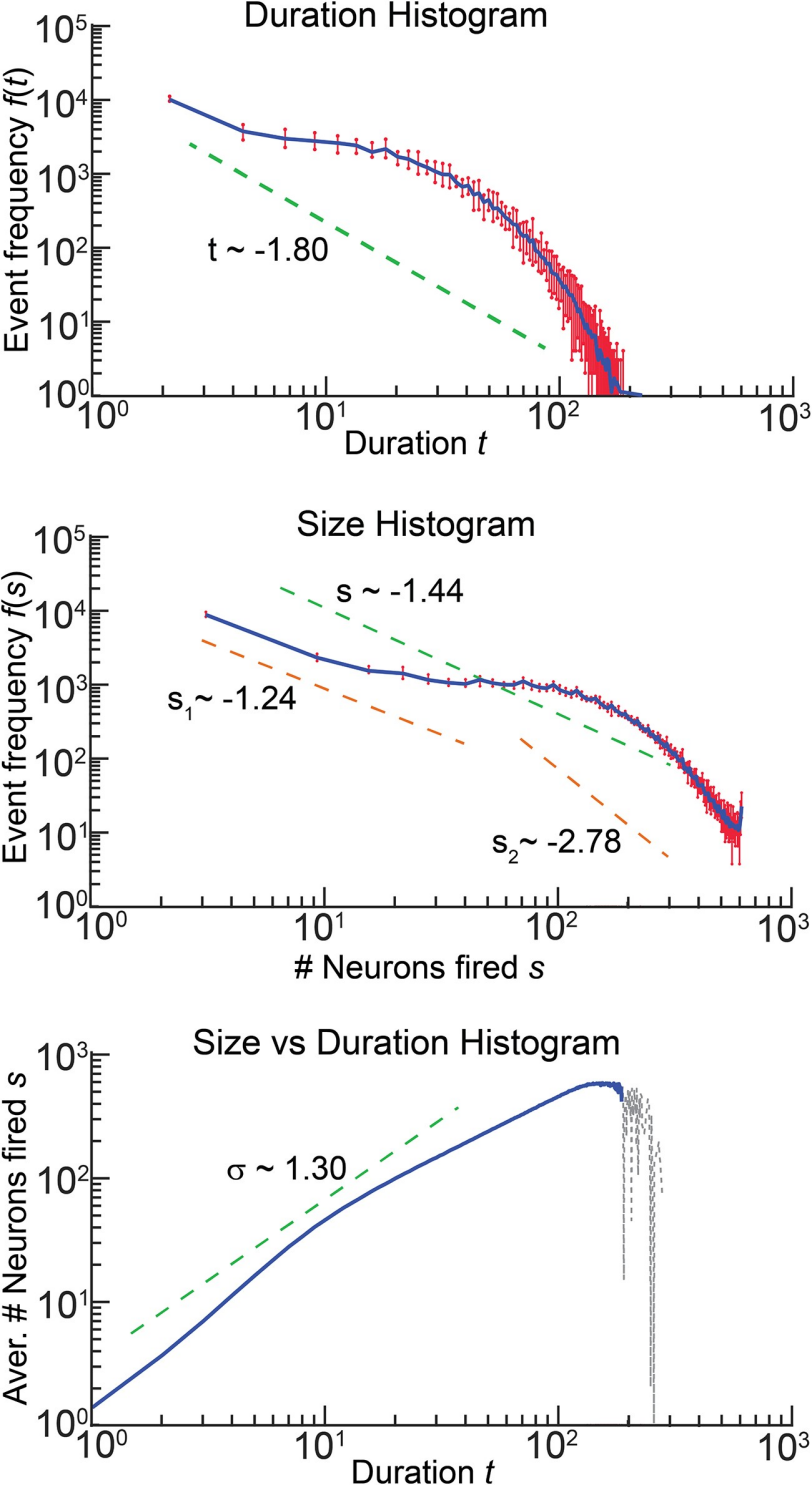
Statistics of sizes and durations of avalanches for *r*_2_ = 5.5, *r*_1_ = 1.1, and their estimated exponents. Red bars indicate intervals within which the values of empirical frequencies varied over 10 different simulations. Blue curves show empirical means. Dashed gray curve in the bottom plot shows a part of the curve corresponding to events for which the data was limited to just few records.

We simulated forward orbits of model (10) for various initial conditions and parameter values, and determined sizes and durations of avalanches of firing events. In our experiments, the avalanches were defined as events corresponding to the intervals *T*_*j*_ = [*t*_*j*_, *t*_*j*+1_] of the network nonzero firing activity such that $\sum_{i=1}^{N}q_{i,t}>0$ for all *t* ∈ [*T*_*j*_] and $\sum_{i=1}^{N}q_{i,t_{j}-1}=\sum_{i=1}^{N}q_{i,t_{j+1}+1}=0$. Each orbit was simulated for 10^6^ time steps, with *q*_*i*,0_ = 0, *i* = 1, …, *N* and *E*_*i*,0_, *i* = 1, …, *N* chosen randomly in the interval $\left[0.5\underline{E},\bar{E}\right]$. For each orbit, we gathered statistics of sizes and durations of the observed avalanches. A brief summary of these experiments is shown in Figs 9 and 10. Fig 9 presents size and duration histograms as a function of parameters *r*_1_ and *r*_2_ in (10). As we can see from this figure, energy feedback has the capacity to inhibit system-size events in networks sharing the same graph topologies, and parameters of this feedback may affect the exponents of size and duration histograms. In particular, we observed that increasing the values of *ε* lead to increases of system-size events and pushing the system eventually into the super-critical state.

Fig 10 shows statistics and estimated exponents of avalanches for *r*_2_ = 5.5, *r*_1_ = 1.1. The estimated exponents are close to those reported for live neuronal cultures [7], [8], [39]. Observe that the size and duration curves have noticeable humps (cf. [39], Fig 2; [40], Figs 3–6), albeit different and less prominent exponents than those reported in [39].

Activity of individual nodes, as a function of *t*, appeared to be synchronized with the peaks of their corresponding energy variables. Similar dependency has been observed in the mean-field approximation too. These observations suggest that exogenous energy may be a relevant factor in understanding dynamics of neural networks. Complementary to connectivity-activity relations revealed in e.g. [17], [23], here we show that energy balance may modulate dynamics of activity patterns in the network. Indeed, in our experiments the network was fully connected and as such its connectivity was always above the network’s percolation threshold. Yet, as Fig 9 illustrates, occurrences and parameters of system-size events in such systems can be controlled by energy balance within individual nodes. This gives rise to functional dynamical clustering in the model, as opposed to clustering induced merely by network connectivity.

### Modelling dynamics observed in neuronal cultures

The mean-filed and multi-agent models introduced and investigated in the previous sections show a range of behaviors which are controlled by just few parameters. These parameters, in turn, may be related to physical quantities and variables such as the connectivity density parameter, thresholds, energy balance parameters (costs of spike generation and transmission), and energy recovery times. Figs 6–8 (see also the online repository [41] containing extended simulation results) show different dynamical modes and behaviors corresponding to these parameters and their combinations. For a broad range of parameters (red areas in Figs 6–8), dynamics of the activity variable *q*_*t*_ in (4) bears qualitative similarity to some of the patterns observed in evolving neuronal cultures at different development stages [42], [43]: tiny bursts, fixed size bursts, variably sized bursts, and superbursts.

**Fig 11:**
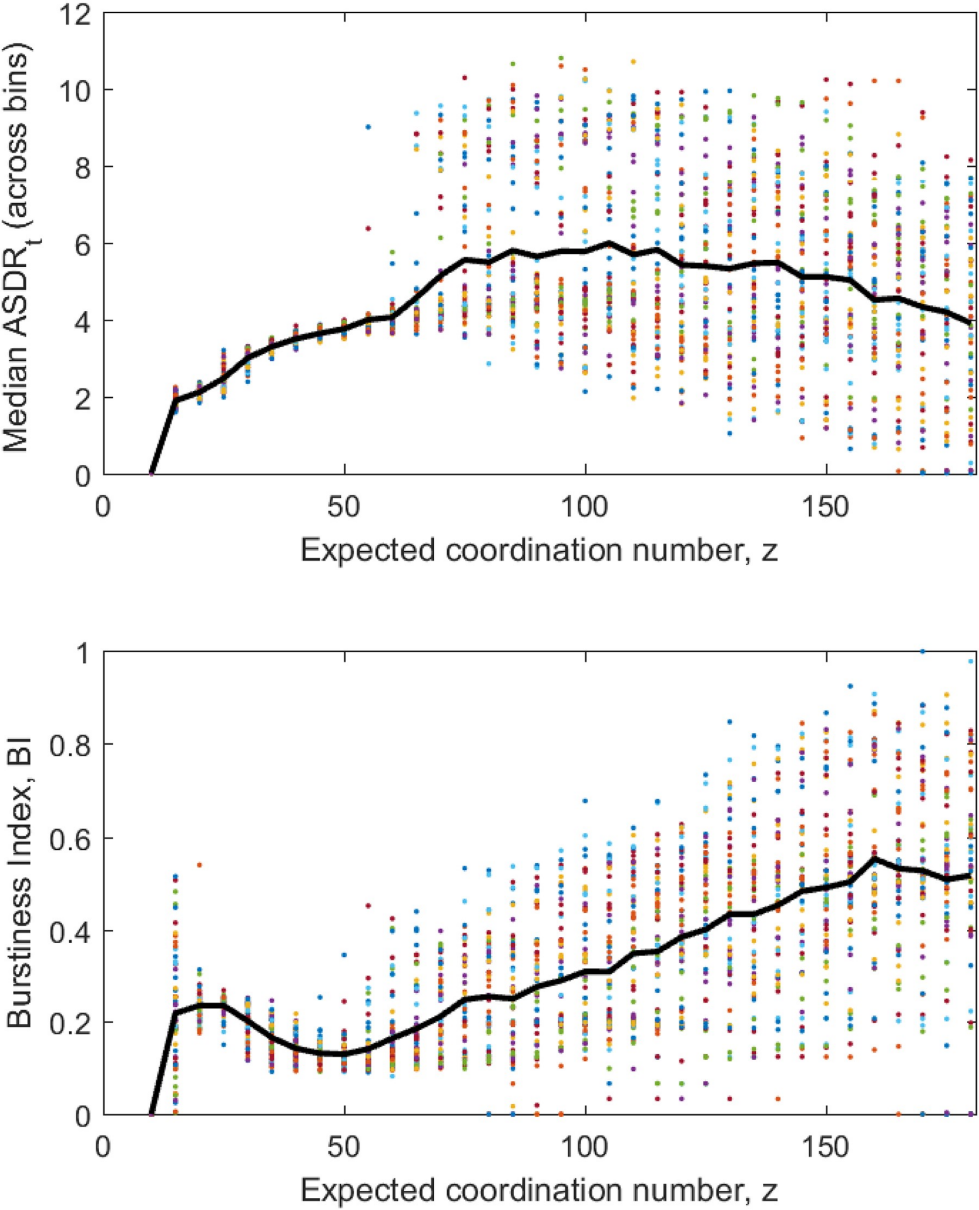
Firing and bursting activity of (4) as a function of *z*. *Top panel* shows the values of median ASDRs calculated across 1000 of adjacent and non-overlapping intervals of *t* (see Methods), each containing *k* = 30 elements. *Bottom panel* shows the values of BI (see Methods). The values of median ASDRs and BIs derived for the same values of *p*, *r*, and $\underline{E}$ are shown in the same color. Black lines show sample averages.

In addition to patterns in model trajectories, we looked at statistics of the Array-wide Spike Detection Rates (ASDR) and Burstiness Indexes (BI) over various days in development (DIV) [42] and compared it with that of the values of ASDR and BI derived from the data generated by our model (see Methods for details). To relate empirical observations to our model, we assumed that the model coordination number *z* is representative of DIV for cultures. Results are shown in Fig 11.

Similarly to how the median ASDRs change in real cultures with DIV (Fig 4, Panel A, [42]), the sample average of the model’s median ASDRs first grows and then decreases with the growth of the model connectivity parameter *z*. Note that the initial growth phase is in agreement with the supercritical Neimark-Sacker bifurcation of the stable equilibrium of (4) (Proposition 2 and discussion afterwards). Moreover, the sample average of the model’s BI as a function of *z* (Fig 11, bottom panel) shows a development trend that is very similar to the one observed in real neuronal cultures (Fig 4, Panel B, [42]).

Last but not least, the proposed model enables explicit modelling of the influence of external metabolic factors on the overall activity in the cultures. This is an advantage over networks of classical conductance-based models like e.g. Hodgkin-Huxley, Hindmarsh-Rose, and Fitzhugh-Nagumo equations [44].

To demonstrate this possibility we modelled the network’s activity during and after acute but short oxygen deprivation as reported in [45]. In [45], primary cultures of hippocampal cells were subjected to 10min of acute oxygen deprivation in the 21st DIV. Reoxygenation after short-term hypoxia rapidly restores energy deficit and neuronal ATP levels and increases the release of glutamate. Glutamate is a major excitatory neurotransmitter in mammalian central nervous system. Glutamatergic neurons form the main excitatory system in the brain and play a pivotal role in many neurophysiological functions [46]. Excessive glutamate releasing is homeostatic response to the hypoxia-induced network silence [47]. It is directed at restoration of network activity and results in an over-activation of ATP-dependent ion pumps and bioenergetic-dependent network burst. However, excessive glutamate releasing over-activates its receptors and changes calcium homeostasis that in turn leads to a cascade of intracellular events causing neuronal degeneration and referred to as excitotoxicity [48], [49].

**Fig 12:**
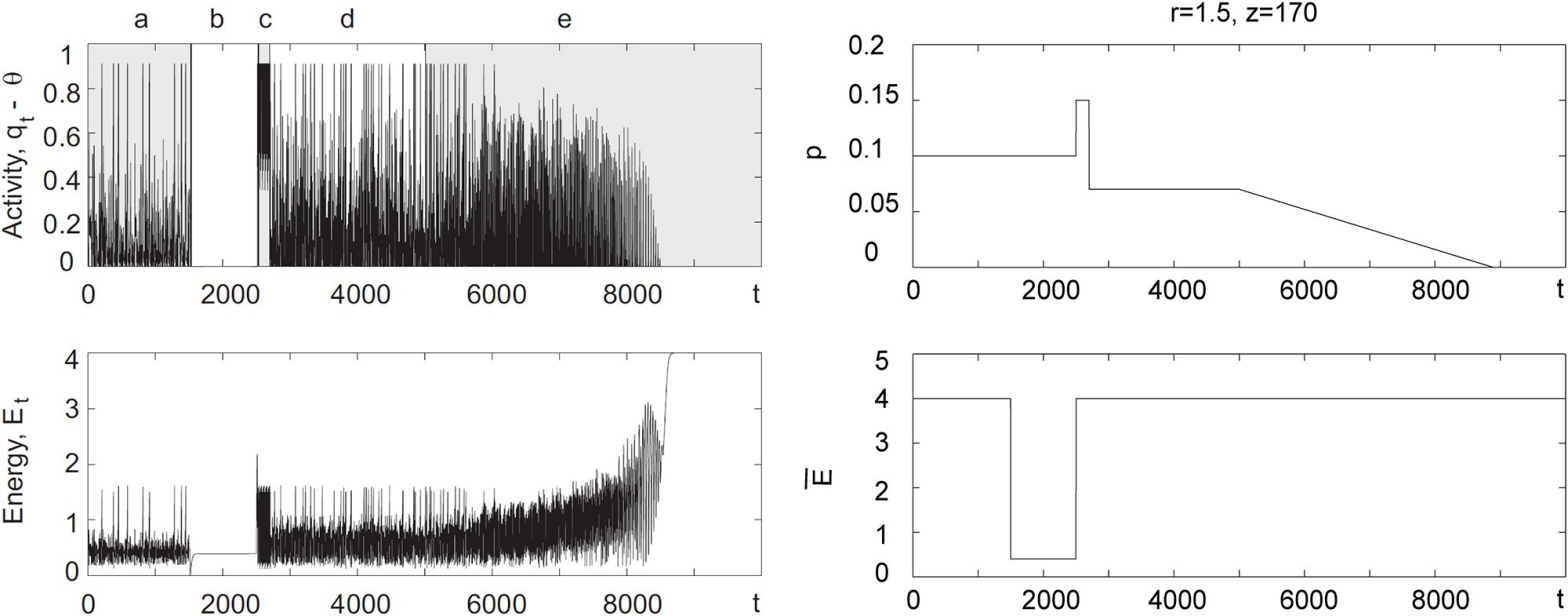
Behavior of the model with phenomenological simulation of the effect of acute oxygen deprivation. Zone *a* in the left plot corresponds to normal functioning, *b* shows effect of acute hypoxia, *c* emulates glutamate induced increase of activity, *d* and *e* are the intervals corresponding to glutamate-induced suppression of neurons and degenerative damage.

To simulate, albeit qualitatively, changes in firing dynamics caused by the hypoxia as well as by the sequence of complex biological of changes related to oxygen deprivation, the following numerical experiments we performed. The model, Eq (4), with *r* = 1.5, *z* = 170, *ε* = 0.05, *w* = 1.5, $\underline{E}=2$, $\bar{E}=4$, *p* = 0.1 was iterated for 1500 steps. Then for the next 1000 steps the value of $\bar{E}$ was reduced to 0.4 and then restored back to the nominal level of 4 for *t* > 2500. This modelled energy deficit caused by acute oxygen deprivation. At *t* = 2500, however, the value of *p* was increased to 0.15 to account for glutamate release, and then dropped to the level *p* = 0.07 in the interval [2700, 5000] to emulate glutamate-induced suppression [50]. For *t* > 5000 the value of *p* was made to decrease linearly to account for degenerative processes triggered by oxygen deprivation. Model behaviour as well as the evolution of *p* and $\bar{E}$ over time are shown in Fig 12. Overall activity levels, which the model shows in this regime, are in qualitative agreement with empirical observations reported in [45].

## Materials and methods

### Construction of Figs 6–8

To construct the figures, we run 3 ⋅ 10^5^ iterations of model (4) from 5 random initial conditions for each relevant set of parameters $\underline{E}$, *z*, *r*, whilst keeping the values of $w,p,\bar{E}$, and *ε* constant (*w* = 1.5, *p* = 0.1, $\bar{E}=4$, *ε* = 0.05). The values of parameter $\underline{E}$ where chosen in the equispaced grid of 21 points in the interval [1, 3]. The values of *z* were varying adaptively. In the intervals (0, 50] and (200, 300] these values were taken from equispaced grids with distances between the grid’s nodes being equal to 0.1. In the interval (50, 200] these distances were set to 5. For each set of model parameters and each initial condition, last 2 ⋅ 10^4^ points in each run have been recorded. Points corresponding to orbits from different initial conditions have been collated together (color-coded), plotted in (*q*_*t*_, *E*_*t*_) space and stored as .gif files at [41]. This resulted in circa 32000 images for *r* = 1 and *r* = 1.5, and circa 10000 images for *r* = 10. The resulting figures were visually inspected and classified into orbits converging to a) single equilibrium (5), b) single equilibrium (6) c) single periodic orbit, d) complicated sets like the ones shown in Fig 5, and e) multiple attractors. These were color-coded and mapped onto the relevant parametric domains.

### Construction of Fig 11

To model ASDR and BI data, we generated 60 × 86 orbits of (4) for different values of *z*, *r*, *p*, and $\underline{E}$. The values of *p*, *r*, and $\underline{E}$ were chosen randomly (60 samples in total) in the intervals [1, 0.11], [1.5, 2] and [2, 2.5], respectively. Randomized choice of *p*, *r*, and $\underline{E}$ simulated fluctuations in neuronal excitability and in the energy balance dynamics across cultures; increments of the values of *z* modelled development of the network connectivity with the culture’s age or DIV. Each triple of fixed but randomly chosen *p*, *r*, $\underline{E}$ was assigned 86 equally spaced values of *z* in the interval [10, 180]. Such model parametrization enabled us to simulate an ensemble of neuronal cultures capturing culture-to-culture variability as well as the cultures’ development over time.

For each triple of *p*, *r*, $\underline{E}$ and every *z*, we generated an orbit of (4) comprising of 2 ⋅ 10^5^ iterates, and the orbits’ segments composed of the last 3 ⋅ 10^4^ elements were used in the calculations. For each segment, we calculated the model’s analogue of the ASDR, as well as the model’s BI. The model’s ASDR was defined as

$$\begin{array}{c} {ASDR}_{t}=\left(q_{t}-\theta \right)H\left(q_{t}-\theta \right), \end{array}$$

where the value of threshold *θ* was set to 1/0.6745 of the median of *q*_*t*_ in the segment (cf. [51]). The model’s BI was determined in accordance with the approach presented in [42]. The entire segment of the orbit was partitioned into adjacent and non-overlapping bins, each containing *k* consecutive measurements of *ASDR*_*t*_. In our numerical experiments, we set *k* = 30. For each bin, we calculated the sum of *ASDR*_*t*_ in the bin. This was followed by determining the fraction of the total activity contained in the top *m*% of the bins. This fraction was recorded as the variable *f*_*m*_. The model’s BI was then defined as [42]:

$$\begin{array}{c} \text{BI}=\frac{f_{m}-\frac{m}{100}}{1-\frac{m}{100}}. \end{array}$$

Since the average width of *ASDR*_*t*_ spikes in the model was about 10 steps, the value of *m* was set to 50 (cf. [42]).

## Conclusion

In summary, we have proposed a simple network model explaining burst generation in living culture networks. A distinct feature of our model is presence of a dynamic exogenous energy variable and neuronal activation probability that is made dependent on the energy, like in general models of physiological adaptation [28]. We showed that introduction of these modifications already enables to explain evolution of cultures from resting state to population bursts, at least in the mean-field approximation. In accordance to the model, emergence of bursts and spikes is regulated by just few parameters that correspond to network connectivity and efficacy of synaptic transmission. We also note that our energy-based model is complementary to more traditional connectivity-focused approaches [23].

In this particular study, when comparing empirical data with model behavior, the number of days in development has been related to network connectivity. We note, however, that the latter in a broader biological context, can depend on various external factors such as e.g. stress [52]. The proposed model hence might be able to predict qualitatively the effect of stress and adaptation to stress on neuronal activity.

Large-scale multi-agent simulations demonstrated that these additional variables are capable of governing the network’s dynamical state and keeping it at the edge of percolation transition, depending on parameters. The energy feedback acts as as a mechanism for controlling and maintaining metabolic homeostasis; this enables communication between nodes across the whole network and at the same time prevents network’s overload caused by excessive propagation of activity. The energy feedback suppresses excitation in individual neurons by disabling, in effect, high-frequency local spike generation. It can therefore be also viewed as an implementation of frequency dependent synaptic plasticity [17].

Despite qualitatively explaining certain phenomena observed in neuronal cultures, the model is simplistic. It does not account for varying strengths of synaptic efficacy, plasticity mechanisms and their time scales, and density of cells. It also may be interesting to look at and thoroughly investigate the distributions of neural firing rates for different values of model parameters and relate these distributions to empirical evidence reported in the literature [53]. Accounting for these is the subject of our future work.

## Ethics statement

No animals or human subjects were used or employed as a part of research presented in this work.

## Appendix. Proofs of technical statements

### Proof of Proposition 1

Notice that *a* = (1 − *p*)^*z*^ ∈ (0, 1) for all *p* ∈ (0, 1), *z* > 0. Thus $1-a^{q_{t}}\in [0,1]$ for all *q*_*t*_ ∈ [0, 1], and forward invariance of [0, 1] follows. The right-hand side of (2) is continuous and strictly monotone with respect to *q*_*t*_ on (0, 1], with *q*_*t*_ = 0 being an equilibrium. Hence all forward orbits of this map, i.e. *q*_*t*_, *q*_*t*+1_, *q*_*t*+2_, … are monotone, and map (2) has only fixed points as attractors. Furthermore, the right-hand side of (2) is strictly concave, which implies that the number of fixed points is at most two.

If the value of *p* is such that the right derivative of

$$\begin{array}{c} f\left(q_{t}\right)=1-{\left(1-p\right)}^{zq_{t}} \end{array}$$

**Fig 13:**
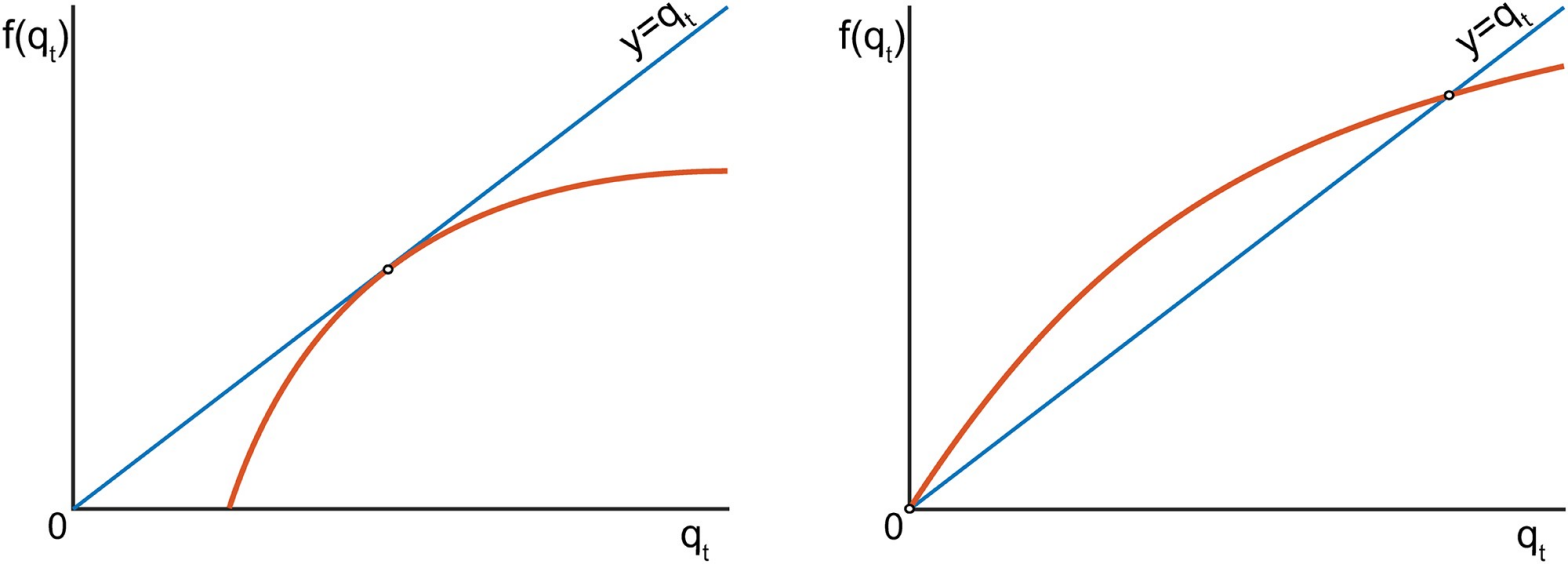
Illustration to the proof of Proposition 1.

*q*_*t*_ = 0 is less or equal to 1 then strict concavity of *f*(⋅) implies that *f*(*q*_*t*_) < *q*_*t*_ for all *q*_*t*_ ∈ (0, 1]. Hence (2) has only one fixed point, *q*_*t*_ = 0. The corresponding condition is −*z* log(1 − *p*) ≤ 1. This fixed point is attracting: lim_*t* → ∞_ *q*_*t*_ = 0. If −*z* log(1 − *p*) > 1 then the trivial equilibrium *q*_*t*_ = 0 becomes a repeller and the second fixed point $q_{t}=\tilde{q}$, $\tilde{q}\in (0,1)$ appears. At this point, the line *y* = *q* and the curve *y* = *f*(*q*) = 1 − (1 − *p*)^*zq*^ intersect transversely. Indeed, if this is not the case then there is a point *q*′ > 0 such that $1-(1-p{)}^{zq^{\prime }}=0$ (see Fig 13, left panel). The latter, however, is impossible as (1 − *p*) ∈ (0, 1). Moreover, at the point of this intersection, the slope of the curve *y* = *f*(*q*) = 1−(1 − *p*)^*zq*^ is always strictly smaller than one (see Fig 13, right panel). In order to see this, recall that the following must hold at $q=\tilde{q}$:

$$\begin{array}{c} \tilde{q}=f\left(\tilde{q}\right)=\int_{0}^{\tilde{q}}\frac{df}{dq}\left(q\right)dq. \end{array}$$

If $df/dq(\tilde{q})\geq 1$ then strict concavity of *f* implies that *df*/*dq*(*q*) > 1 for all $q\in (0,\tilde{q}]$. This, however, contradicts to that $\tilde{q}=f(\tilde{q})$. Hence the slope of the function *f*(⋅) at the second fixed point, $\tilde{q}$, is strictly smaller than one, and the fixed point is locally exponentially stable.

### Proof of Proposition 2

*Forward invariance*. Similar to the proof of Proposition 1, we observe that $0<p\sigma (E_{t},\underline{E})<1$ for all *E*_*t*_. Hence $a=(1-p\sigma (E_{t},\underline{E}){)}^{z}\in (0,1)$ and $1-a^{q_{t}}\in [0,1]$ for all *q*_*t*_ ∈ [0, 1]. This implies that *q*_*t*_ ∈ [0, 1] for all *t* regardless of the values of *E*_*t*_. Let $E_{t}\in [0,\bar{E}]$ and *E*_*t*_ < *rq*_*t*_. In this case, $E_{t+1}=(1-\varepsilon )E_{t}+\varepsilon \bar{E}\in [0,\bar{E}]$. If $E_{t}\in [0,\bar{E}]$ and *E*_*t*_ ≥ *rq*_*t*_, then

$$\begin{array}{c} E_{t+1}=\left(1-\varepsilon \right)E_{t}+\varepsilon \bar{E}-rq_{t}\geq \varepsilon \left(\bar{E}-E_{t}\right)\geq 0\\ E_{t+1}\leq \left(1-\varepsilon \right)\bar{E}+\varepsilon \bar{E}-rq_{t}\leq \bar{E}. \end{array}$$

Thus the domain $\left\{(q,E)\;|q\in [0,1],E\in [0,\bar{E}]\right\}$ is forward-invariant.

*Statement 1*. Observe, that $q_{1}^{*}=0$, $E_{1}^{*}=\bar{E}$ is always an equilibrium of (4). At this equilibrium, $\bar{E}=E_{1}^{*}\geq rq_{1}^{*}=0$, and hence the Jacobian $J(q_{1}^{*},E_{1}^{*})$ of the right-hand side of (4) at this equilibrium is:

$$\begin{array}{c} J\left(0,\bar{E}\right)=(\begin{array}{cc} -z\log(1-p\sigma \left(\bar{E},\underline{E}\right)) & 0\\ -r & 1-\varepsilon \end{array}) \end{array}$$

It is clear that the eigenvalues of $J(0,\bar{E})$ are $\lambda_{1}=-z\;\text{log}(1-p\sigma (\bar{E},\underline{E}))$, λ_2_ = 1 − *ε*. Therefore, the fixed point $\left(q_{1}^{*},E_{1}^{*}\right)$ is an attractor when $-z\;\text{log}(1-p\sigma (\bar{E},\underline{E}))<1$ and is a repeller when $-z\;\text{log}(1-p\sigma (\bar{E},\underline{E}))>1$. This proves statement 1 of the proposition.

*Statements 2,3*. Let (q*, E*) with q* ≠ 0 be another equilibrium of (4). All such equilibria of (4) must satisfy

$$\begin{array}{c} \begin{array}{cc} q^{*}= & 1-{\left(1-p\sigma \left(E^{*},\underline{E}\right)\right)}^{q^{*}z}\\ E^{*}= & \left(1-\varepsilon \right)E^{*}+\varepsilon \bar{E}-rq^{*}H\left(E^{*}-rq^{*}\right). \end{array} \end{array}$$

Depending on the sign of E* − rq*, the above system splits into the following two cases:

$$\begin{array}{c} \left\{ \begin{array}{cc} q^{*}= & 1-{\left(1-p\sigma \left(E^{*},\underline{E}\right)\right)}^{q^{*}z}\\ E^{*}= & \bar{E}-\frac{rq^{*}}{\varepsilon },\;\text{if}\;E^{*}\geq rq^{*}, \end{array}\;\{\begin{array}{cc} q^{*}= & 1-{\left(1-p\sigma \left(E^{*},\underline{E}\right)\right)}^{q^{*}z}\\ E^{*}= & \bar{E},\;\text{if}\;E^{*}<rq^{*}. \end{array}\right. \end{array}$$ (11)

Let $g(\cdot )=\sigma (\cdot ,\underline{E})$. Note that the function g(⋅):ℝ→(0,1) is continuous and strictly increasing for *w* > 0. Hence $g^{-1}(\cdot ):(0,1)\to \mathbb{R}$ exists, and is continuous and strictly increasing too. Moreover, points (q*, E*) satisfying (11) should satisfy conditions below (and vice-versa):

$$\begin{array}{c} \left\{ \begin{array}{cc} E^{*}= & g^{-1}(\frac{1}{p}(1-{\left(1-q^{*}\right)}^{\frac{1}{q^{*}z}})),\\ E^{*}= & \bar{E}-\frac{rq^{*}}{\varepsilon },\;\text{if}\;E^{*}\geq rq^{*}, \end{array}\;\{\begin{array}{cc} E^{*}= & g^{-1}(\frac{1}{p}(1-{\left(1-q^{*}\right)}^{\frac{1}{q^{*}z}})),\\ E^{*}= & \bar{E},\;\text{if}\;E^{*}<rq^{*}. \end{array}\right. \end{array}$$ (12)

Consider the functions

$$\begin{array}{c} \begin{array}{cc} h(q^{*})= & g^{-1}(\frac{1}{p}(1-{\left(1-q^{*}\right)}^{\frac{1}{q^{*}z}})),\\ f(q^{*})= & {\left(1-q^{*}\right)}^{\frac{1}{q^{*}}}. \end{array} \end{array}$$

Let Ω be the domain of the definition of the function *h*(⋅). If a solution of (12) exists with q* ∈ (0, 1) then the intersection

$$\begin{array}{c} \left(0,\bar{q}\right)=\Omega \cap \left(0,1\right) \end{array}$$

must be non-empty. The function *f*(⋅) is continuous on (0, 1), and its first derivative,

$$\begin{array}{c} \begin{array}{cc} & \frac{d}{dq^{*}}f={\left(1-q^{*}\right)}^{\frac{1}{q^{*}}}(-\frac{1}{{q^{*}}^{2}}\log\left(1-q^{*}\right)-\frac{1}{q^{*}\left(1-q^{*}\right)})\\ & =-\frac{1}{{q^{*}}^{2}}{\left(1-q^{*}\right)}^{\frac{1}{q^{*}}}(\log\left(1-q^{*}\right)+\frac{q^{*}}{1-q^{*}}), \end{array} \end{array}$$

is strictly negative in (0, 1). This implies that the function *h*(⋅) is strictly increasing in $\left(0,\bar{q}\right)$.

Consider the case when E* ≥ rq*, (left system in (12)). Given that $E^{*}=\bar{E}-rq^{*}/\varepsilon$, as a function of q*, is strictly decreasing on (0, 1), and *h*(⋅) is strictly increasing on $\left(0,\bar{q}\right)$, there must be at most one equilibrium of (4) in $\left(0,\text{min}\{1,\bar{q}\})âŠ‚(0,1\right)$. If such equilibrium exists then it must satisfy

$$\begin{array}{c} q^{*}=1-{\left(1-p\sigma (\bar{E}-\frac{r}{\varepsilon }q^{*},\underline{E})\right)}^{zq^{*}} \end{array}$$ (13)

for some q* ∈ (0, 1). This, however, is possible only if the the derivative of the right-hand side of (13) at q* = 0 is larger than 1. The corresponding condition is

$$\begin{array}{c} \begin{array}{cc} & \frac{d}{dq^{*}}(1-{\left(1-p\sigma (\bar{E}-\frac{r}{\varepsilon }q^{*},\underline{E})\right)}^{zq^{*}})=-\frac{d}{dq^{*}}({\left(1-p\sigma (\bar{E}-\frac{r}{\varepsilon }q^{*},\underline{E})\right)}^{zq^{*}})=\\ & -{\left(1-p\sigma (\bar{E}-\frac{r}{\varepsilon }q^{*},\underline{E})\right)}^{zq^{*}}{|}_{q^{*}=0}\times \\ & \times [z\log(1-p\sigma (\bar{E}-\frac{r}{\varepsilon }q^{*},\underline{E}))+\frac{zq^{*}p}{1-p\sigma (\bar{E}-\frac{r}{\varepsilon }q^{*},\underline{E})}\frac{d}{dq^{*}}\sigma (\bar{E},\underline{E})]{|}_{q^{*}=0}\\ & =-z\log(1-p\sigma (\bar{E},\underline{E}))>1. \end{array} \end{array}$$

Consider the case when E* < rq* (the right system in (12)). Equilibria corresponding to this alternative must satisfy $q^{*}>\bar{E}/r$. This, however, is possible only if $\bar{E}/r<1$. The alternative condition, $\bar{E}/r\geq 1$, therefore implies that E* ≥ rq*. The latter observation completes the proof of statement 2.

Let $\bar{E}/r<1$ (or E* < rq*). Given that the function *h*(⋅) is strictly monotone on $\left(0,\text{min}\{1,\bar{q}\}\right)$, only one equilibrium with $E^{*}=\bar{E}$ may exist. At this equilibrium,

$$\begin{array}{c} z\log(1-p\sigma (\bar{E},\underline{E}))=\frac{\log(1-q^{*})}{q^{*}}<-1 \end{array}$$

and

$$\begin{array}{c} |\frac{d}{dq^{*}}\left(1-{\left(1-p\sigma \left(\bar{E},\underline{E}\right)\right)}^{q^{*}z}\right)|<1. \end{array}$$

The latter inequality is due to that $1-(1-p\sigma (\bar{E},\underline{E}){)}^{q^{*}z}$ is strictly concave with respect to q* on (0, 1) (see the proof of Proposition 1). Consider the Jacobian $J(q^{*},\bar{E})$:

$$\begin{array}{c} J\left(q^{*},\bar{E}\right)=(\begin{array}{cc} \frac{d}{dq^{*}}\left(1-{\left(1-p\sigma \left(\bar{E},\underline{E}\right)\right)}^{q^{*}z}\right) & \left[*\right]\\ 0 & \left(1-\varepsilon \right) \end{array}), \end{array}$$

where [*] stands for the corresponding entry of the Jacobian matrix. The absolute values of its eigenvalues are clearly less than 1, and hence the fixed point is a stable attractor.
